# The interactions of Pu22 G-quadruplex, derived from *c-MYC* promoter sequence, with antitumor acridine derivatives—An NMR/MD combined study

**DOI:** 10.1016/j.omtn.2025.102513

**Published:** 2025-03-13

**Authors:** Tomasz Laskowski, Michał Kosno, Witold Andrałojć, Julia Pakuła, Rafał Stojałowski, Julia Borzyszkowska-Bukowska, Ewa Paluszkiewicz, Zofia Mazerska

**Affiliations:** 1 Department of Pharmaceutical Technology and Biochemistry, Faculty of Chemistry, Gdańsk University of Technology, Gabriela Narutowicza Str. 11/12, 80-233 Gdańsk, Poland; 2 Institute of Bioorganic Chemistry, Polish Academy of Sciences, Zygmunta Noskowskiego Str. 12/14, 61-704 Poznań, Poland

**Keywords:** MT: Oligonucleotides: Therapies and Applications, antitumor agents, nuclear magnetic resonance, NMR, acridine derivatives, molecular modeling, DNA G-quadruplexes, drug-DNA interactions, Symadex, nitracrine

## Abstract

Unsymmetrical bisacridines (UAs) represent a novel class of anticancer agents that exhibit significant antitumor activity against a wide range of cancer cell lines and solid tumors *in vivo*. UAs consist of two different acridine-based ring systems, which are connected by an aminoalkyl linker. Recent studies have demonstrated that UAs can suppress the *c-MYC* protooncogene, which is overexpressed in many tumor types. As a proposed molecular basis for this activity, UAs have been suggested to stabilize the G-quadruplex structure formed within the promoter region of *c-MYC*. In this study, we performed spectroscopic and computational analyses to investigate the stereochemistry of the *c-MYC* NHE III_1_ representative G-quadruplex, codenamed Pu22, in complex with two promising bisacridines, C-2045 and C-2053, as well as their monomeric counterparts, C-1311 and C-1748. C-1311 formed a well-defined 1:2 mol/mol DNA:ligand non-covalent adduct, whose solution structure was determined via 2D NMR. In contrast, C-1748 displayed weak and nonspecific interactions with the Pu22 G-quadruplex. Finally, the Pu22:UA complexes were examined using a combination of NMR and molecular modeling approaches, including umbrella sampling simulations. These results provide insights into the interaction mechanisms of UAs with G-quadruplex structures and highlight their potential as therapeutic agents targeting *c-MYC*.

## Graphical abstract

**Figure:**
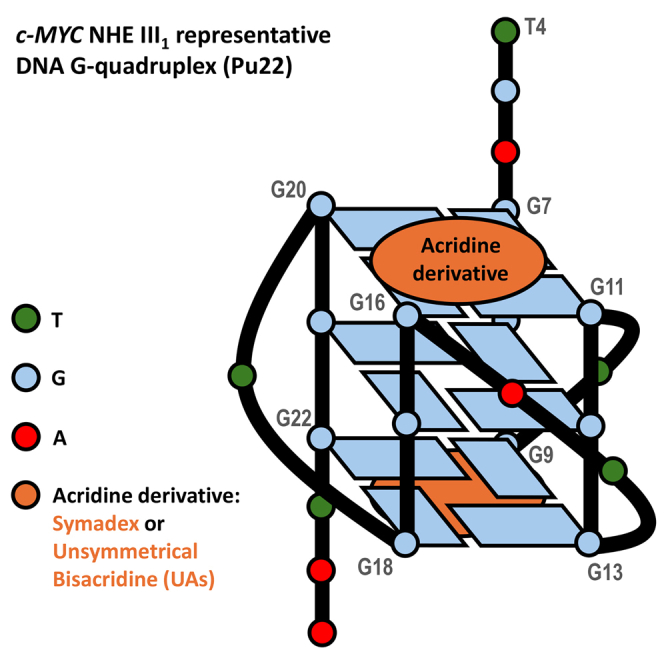

## Teaser

Unsymmetrical bisacridines (UAs) are novel anticancer agents that suppress the c-MYC protooncogene by stabilizing G-quadruplex structure formed in its promoter region. Nuclear magnetic resonance and computational studies examined their interaction with c-MYC’s G-quadruplex, revealing specific binding by UAs and their monomeric subunit—C-1311.

## Introduction

Unsymmetrical bisacridines (UAs) are a novel class of anticancer compounds exhibiting high antitumor activity against numerous cancer cell lines, including pancreatic cancer cell cultures.^1^ To this date, 10 human tumor xenografts in nude mice responded well while exposed to UAs.^1^ They are highly cytotoxic compounds, displaying half maximal inhibitory concentration (IC_50_) values at ng/mL level, although individual cell lines may respond differently to the UA species.^1^^,^^2^ Previous results established that cells treated with UAs undergo apoptosis or senescence.^2^ The cellular uptake of unsymmetrical bisacridines is rapid, as they are detected inside the cells just after 1 h after the exposure.^3^ Interestingly, UAs tend to accumulate in those organelles, which is characterized by lower pH, such as lysosomes and endosomes.^3^^,^^4^

The molecular mechanism of action of UAs, as well as their molecular targets, are still extensively researched. Recently, a strong inhibition of *c-MYC* protooncogene in HCT116 colorectal cancer and its full inhibition in H460 lung cancer cells was discovered as a response for the cell treatment by the members of the UA family.^5^ *MYC* is an important family of genes involved in transcription process and is responsible for regulation of many physiological processes, like protein synthesis, cell cycle control, cell adhesion, or apoptosis.^6^ *C-MYC* is an important part of the *MYC* family that encodes the *c-MYC* transcription factor, which was for the first time identified as the cellular homolog of the viral oncogene (*v-MYC*) of the avian myelocytomatosis retrovirus.^7^ According to current knowledge, as many as 20% of human cancers can be associated with the overexpression of *c-MYC* via a myriad of different mechanisms.^6^^,^^8^ Aberrant expression of c-MYC is likely attributable to direct gene alterations, which are associated with tumorigenesis and sustained tumor growth.^9^^,^^10^ The nuclease hypersensitivity element III_1_ (NHE III_1_), also known as Pu27 with 27 base pairs (bp), controls 80%–90% of the transcriptional activity of this gene. This guanine (G)-rich element is located −142 to −115 bp upstream of the P1 promoter and forms a transcriptionally active double helix structure.^11^^,^^12^ However, this fragment exhibits an additional feature: under physiological conditions, its double-stranded DNA form coexists with an alternative conformational species, namely a unimolecular DNA G-quadruplex (G4).^13^

To date, numerous guanine-rich genomic fragments, such as gene promoter regions and telomeric sequences, have been recognized as G-quadruplex-forming sites.^14^^,^^15^ The formation of G4 structures in strategic regions of DNA has been associated with several critical cellular processes, including replication, translation, gene transcription, and the maintenance of genomic stability.^16^ Structurally, an individual G-quadruplex consists of two key components: the core and the loops. The core is composed of guanine tetrads, which are held together by Hoogsteen hydrogen bonds, π-π interactions, and the DNA backbone. Each G-tetrad contains eight hydrogen bonds, and each guanosine residue acts as both a donor and acceptor of two hydrogen bonds. These structures are further stabilized by the presence of small cations, which fit between the G4 planes.^17^ The four corners of the tetrads form G-columns. The most common ‘unimolecular’ G-quadruplexes—those formed by a single DNA strand—can be divided into three major topological types: parallel, antiparallel, and hybrid. The G-columns of these structures are connected by three types of loops: propeller, lateral, and diagonal.^18^ The precise relationship between DNA base sequence and G4 structure remains incompletely understood, as individual polynucleotides are often capable of adopting more than one stable G4 topology. Therefore, the resulting G-quadruplex structure is determined by several factors, including nucleotide sequence, the type of ions embedded in the core (typically K^+^, Na^+^, or NH_4_^+^), ionic strength, pH, and other conditions.^17^ Additionally, as unimolecular G4s formed *in vivo* are part of much longer DNA strands, the regions immediately flanking the 5′ and 3′ ends of a G-quadruplex often contain single-stranded polynucleotide segments, referred to as “G4 sidechains” or “flanking residues.”^19^^,^^20^

The formation of a G-quadruplex within the *c-MYC* promoter region enables the study of direct modulation of *c-MYC* expression using small drug-like molecules that bind to and stabilize the G-quadruplex structure.^8^^,^^21^^,^^22^^,^^23^^,^^24^^,^^25^^,^^26^^,^^27^ A typical G-quadruplex ligand is a planar, polyaromatic molecule, optionally with an attached sidechain, capable of interacting with one or both external G-tetrads. In some cases, the ligand intercalates between an external G-tetrad and the adjacent single-stranded polynucleotide residues, exploiting the so-called “drug-binding pockets.” Conversely, various other interactions between G4 sidechains and ligands are possible, including the formation of DNA-ligand hydrogen bonds resembling purine-pyrimidine base pairing in double-stranded DNA (dsDNA).^28^ While G-tetrad stacking and/or interactions with G4 flanking residues are common ligand binding modes, many molecules interact with G4 structures differently, depending on the G4 sequences, topologies, and ligand structures.^18^ Some ligands, despite their flat, polyaromatic structure, bypass the external G-tetrads entirely, preferring to bind in G-quadruplex grooves and/or loops.^29^^,^^30^^,^^31^ Other G4-targeting molecules consist of smaller heteroaromatic rings connected by short linkers, and these typically bind in the G-quadruplex grooves.^32^^,^^33^ Therefore, while the basic concept of a G4/ligand complex is relatively straightforward, G4 polymorphism and the variety of possible G4-ligand interactions make the stereostructures of G4s complexed with actual drugs or drug candidates rare and challenging to obtain.^20^

**Figure 1:**
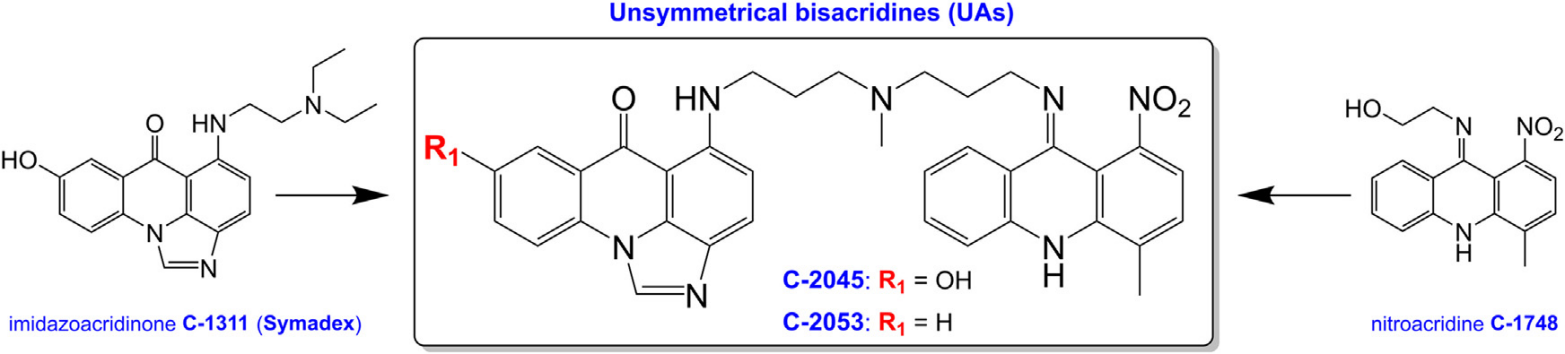
The structures of studied acridine derivatives

As previously mentioned, unsymmetrical bisacridines have been shown to strongly suppress *c-MYC* gene expression, making their potential interactions with the *c-MYC* promoter G-quadruplex worth exploring.^1^ UAs consist of two heteroaromatic ring systems: one forming the imidazoacridinone moiety and the other being a 9-amino-1-nitroacridine derivative (Figure 1).^1^ The former serves as the structural basis for Symadex (C-1311),^34^^,^^35^^,^^36^ which reached Phase II clinical trials,^37^ while the latter is a direct descendant of Nitracrine/Ledakrin (C-283), the first Polish anticancer drug.^38^^,^^39^^,^^40^^,^^41^ These two monomers are connected by an aminoalkyl linker, which can vary in structure. In theory, such molecules can interact with G-quadruplexes in several ways due to their two polyaromatic subunits.

**Figure 2:**
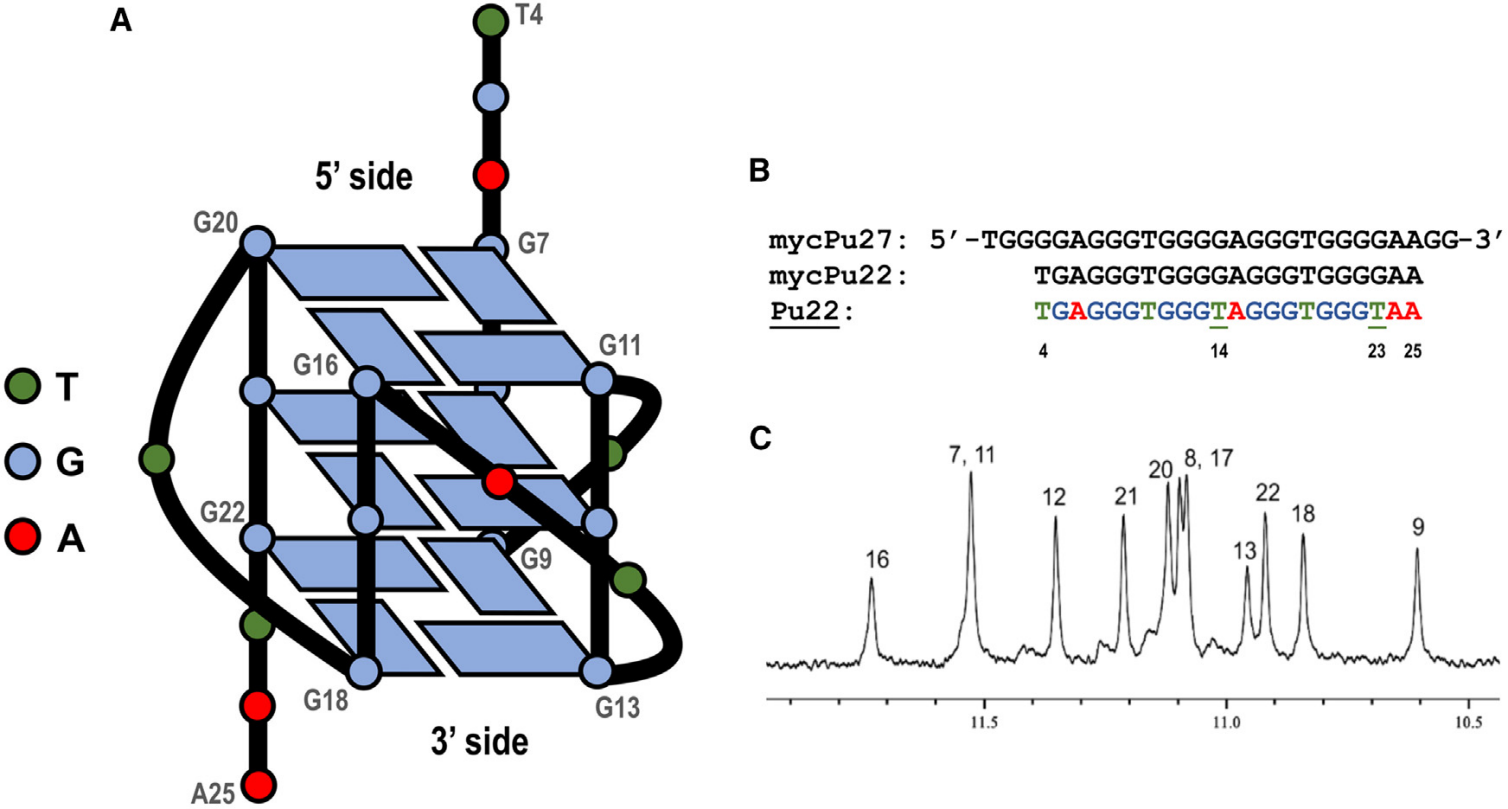
A general structure of the Pu22 DNA G-quadruplex (A) The general structure of Pu22 G-quadruplex. (B) The sequence of mycPu27, originating from promoter sequence of *c-MYC* protooncogene, along with its derivatives: mycPu22 and Pu22. Underlined T14 and T23 positions within Pu22 sequence denote two G→T mutations, transforming mycPu22 into Pu22. (C) ^1^H NMR assignment of imino proton resonances produced by G-tetrads of Pu22, recorded at 50°C, H_2_O/D_2_O 9:1 v/v, pH = 5.0, 10-mM potassium cacodylate buffer with 10 mM KCl.

In this study, we examined the interactions of UAs with the *c-MYC* promoter G-quadruplex using advanced nuclear magnetic resonance (NMR) techniques, supported by molecular modeling computations. Our approach follows the methods previously described by Ambrus et al.^42^ and Dai et al.^20^ To ensure that the G-quadruplex adopted a single dominant conformation in solution, thus simplifying spectroscopic investigations while maintaining biological relevance, we did not examine the mycPu27 sequence encoded in the “wild-type” *c-MYC* promoter region. Previous studies have shown that the mycPu22 fragment, a shorter and slightly modified version of mycPu27 (Figure 2B), forms the major *c-MYC* NHE III_1_ G-quadruplex in physiologically relevant K^+^ solution. However, since mycPu22 still exhibits conformational polymorphism,^42^ we introduced two G-to-T mutations at positions 14 and 23, resulting in the Pu22 sequence, which adopts a single predominant *c-MYC* promoter G-quadruplex (MYC-G4) topology in K^+^ solution (Figure 2A).^20^^,^^42^

Thus, the Pu22 sequence serves as a model MYC-G4 structure, which in this work was targeted by two promising UAs, C-2045 and C-2053, as well as their monomeric counterparts: imidazoacridinone C-1311 (Symadex) and 9-amino-1-nitroacridine C-1748.^43^ The aim of this study was to determine whether these acridine derivatives can form stable, non-covalent complexes with the Pu22 G-quadruplex, and if so, to elucidate the structures of the resulting adducts and identify the structural elements of the ligands that dictate their binding mode to MYC-G4. Understanding the interactions between the *c-MYC* G-quadruplex and these mono- and bisacridines provides important insights into the molecular basis of their antitumor mechanism of action.

## Results

### Reference spectra of Pu22 G-quadruplex

The NMR-derived three-dimensional structure of Pu22 has been reported previously (Figure 2A).^20^^,^^42^ In this report, we have followed the numbering of nucleotides constituting the discussed G-quadruplex as established before (Figure 2B).^20^ While the earlier studies on the creation of Pu22:ligand non-covalent adducts were conducted at pH = 6 or pH = 7, we chose 10 mM potassium cacodylate buffer of pH = 5.0 (with the addition of 10 mM KCl) as a standard environment for NMR experiments (Figure 2C). This decision was based on the complex acid-base equilibrium of the studied unsymmetrical bisacridines and is discussed in the following subsection.

Figure S1 illustrates the sequential assignment procedure, commonly referred to as the “NOESY walk,” for the Pu22 sequence. Proton signals were assigned based on the principle that the H1′ proton of a given deoxyribose moiety exhibits dipolar coupling to the H6/H8 proton of its corresponding nitrogenous base and to the H6/H8 proton of the 3′-adjacent nucleotide. The NOESY walk was interrupted at the T10, T14-A15, and T19 loops, but it still facilitated the assignment of the remaining deoxyribose protons (Figures S2 and S3), which was achieved through NOESY, TOCSY, and ^1^H-^13^C HSQC experiments, as reported in Table S1.

### DNA:ligand complexes formed via titration of Pu22 with acridine derivatives

**Figure 3:**
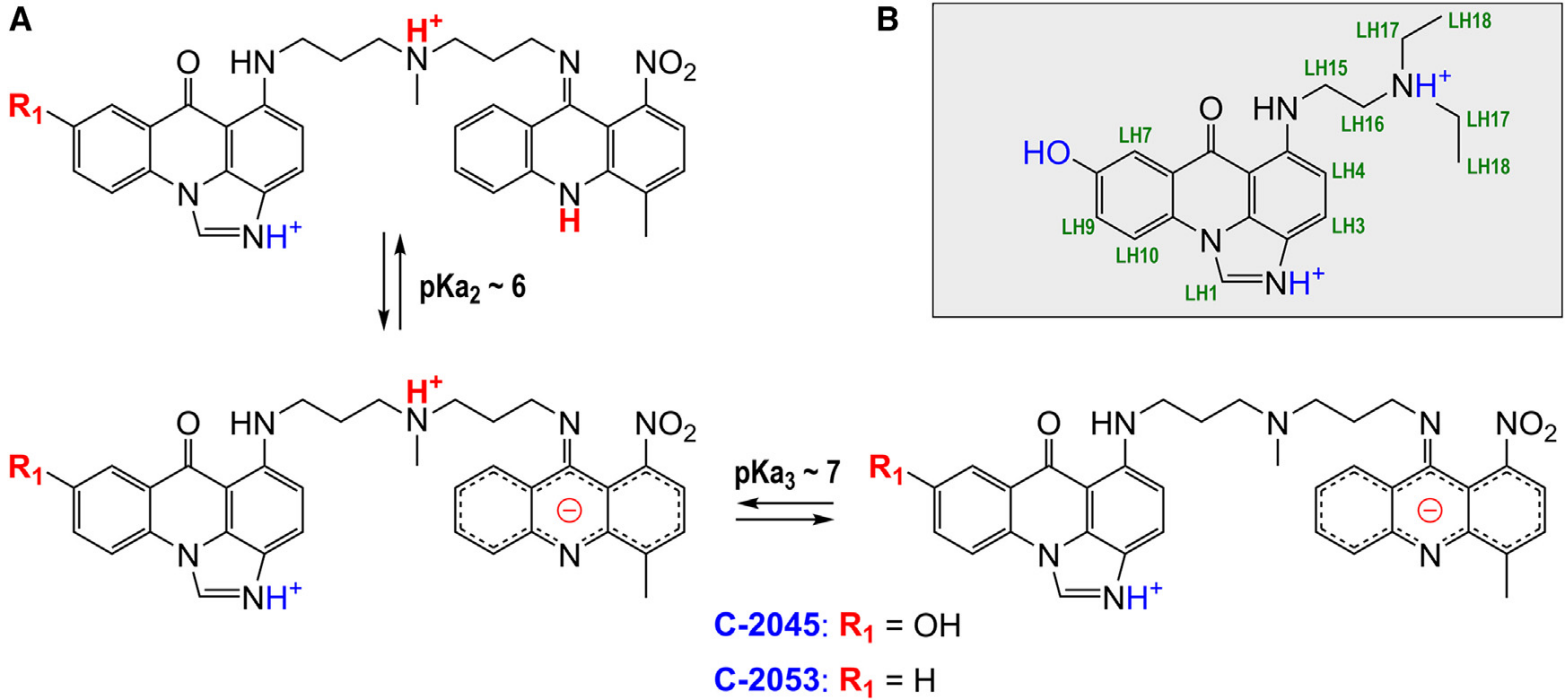
Acid-base equilibrium of the studied acridine derivatives (A) pH-dependent protonation forms of UAs. (B) The structure of C-1311 (Symadex) at pH = 5.0, along with the proton labeling.

When the pH of a solution is set to physiologically relevant values, unsymmetrical bisacridines coexist in three major protonation forms. These forms exhibit different net electric charges and—consequently—solubility, electronic properties, and self-association potential. This effectively makes NMR studies on the stereochemistry of Pu22 complexes impossible at pH = 6 and above. Therefore, we decided to lower the pH to 5.0 in order to isolate a single dominant spectral form of UAs. At pH = 5.0, the net charge of UAs is +2, which strengthens their affinity for negatively charged nucleic acids while reducing the tendency to self-associate at lower concentrations (Figure 3A). At pKa ∼6, the electronic properties of the 9-amino-1-nitroacridine moiety drastically change, whereas at pKa ∼7, the nitrogen atom of the aminoalkyl linker is deprotonated, and the net charge of the molecule changes to 0. Importantly, the protonation of the imidazoacridinone moiety remains unaltered until pH ∼8 is reached. The full scheme of the acid-base equilibrium of unsymmetrical bisacridines, along with the exact pKa values for each individual UA, can be found in our previous manuscript.^44^

**Figure 4:**
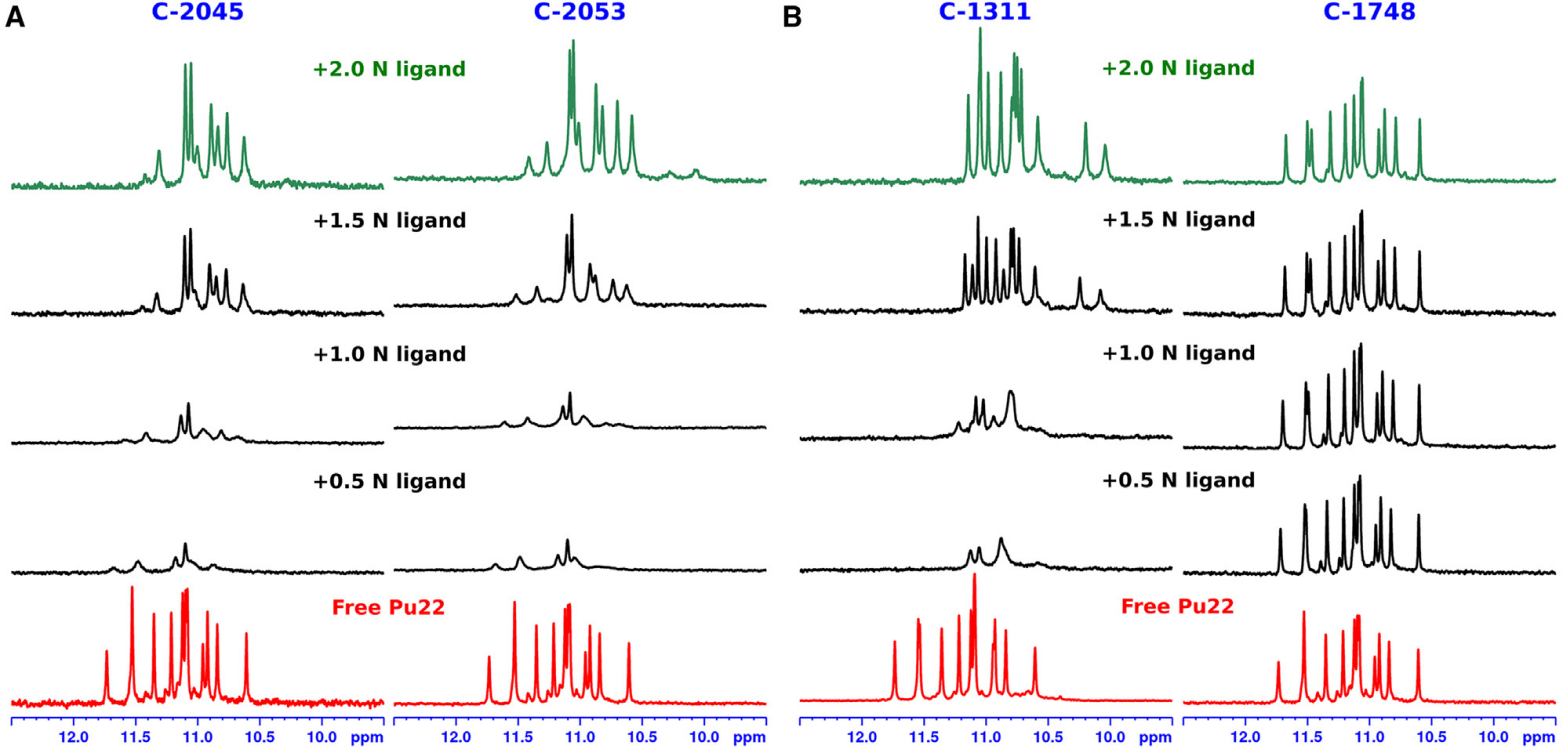
The titration of Pu22 with the studied acridine derivatives ^1^H NMR spectra of Pu22:ligand complexes, depicting imino proton resonances changing upon the titration of DNA with bisacridine ligands (A) and monoacridine ligands (B). Spectra were collected at pH = 5.0, temperature = 50°C; H_2_O/D_2_O 9:1 v/v in 10 mM cacodylate buffer with 10 mM KCl.

Initially, Pu22 was titrated with the two most promising bisacridine derivatives, namely C-2045 and C-2053. 1D ^1^H NMR was chosen as a tool to monitor this process. The resonances of the imino protons gradually shifted as the titrations progressed (Figure 4A). The largest shifts were observed for the signals representing the imino protons bound to the 3′ and 5′ external G-tetrads. The signals corresponding to the protons of the middle tetrad barely shifted at all, whereas their width notably changed during the titration. Very similar changes were observed for both C-2045 and C-2053. Ultimately, the results strongly indicated that both C-2045 and C-2053 interacted with Pu22 in a DNA ratio of 1:2 (Figure 4A). These interactions were relatively specific and strong, revolving around interactions between the planar fragments of UAs and the two external Pu22 G-tetrads.

Based on these findings, NOESY spectra were recorded for both complexes. Despite multiple measurements under varying pH and temperature conditions, either no UA resonance signals were visible in the NOESY spectra, or only weak intermolecular correlations from the imidazoacridinone subunit were detectable. The broadening of the UA ligand resonances indicated that the complexes adopted at least two conformations, which were in a medium exchange regime on the NMR timescale. To overcome this problem and gain insight into the structures of the resulting complexes, we exploited the fact that UAs consist of two aromatic moieties that are not electron-coupled to each other. Both of these rings can be approximated by a monomeric acridine analogue, whose aromatic systems are identical to those of the UA rings: C-1311 (Symadex), which is a monomeric imidazoacridinone fragment, and C-1748, which constitutes the 9-amino-1-nitroacridine moiety. Hence, these two compounds were employed for further studies (Figure 1).

**Figure 5:**
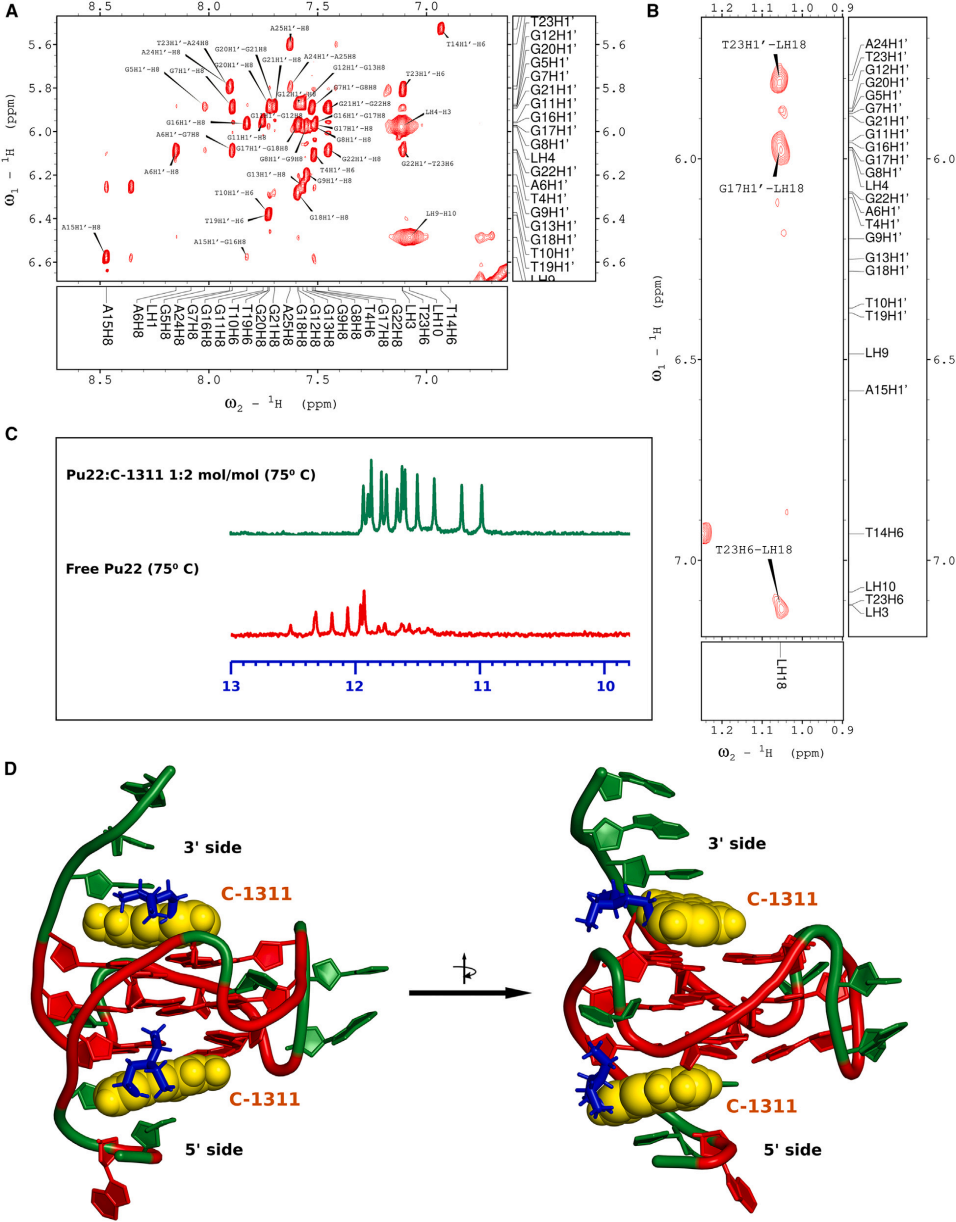
NMR studies on the Pu22:C-1311 1:2 (mol/mol) non-covalent adduct (A) The expanded H8/H6-H1^’^ region of the 2D-NOESY spectrum of the Pu22:C-1311 complex LH4-H3 and LH9-H10 are ligand correlation signals. (B) Exemplary DNA:ligand intermolecular dipolar couplings, produced by the sidechain of C-1311. (C) Thermal stabilization of Pu22 G-quadruplex by two C-1311 molecules. External G-tetrads of the free DNA are almost denatured at 75°C, whereas at the same temperature the DNA:ligand complex remains exceptionally stable. All DNA imino protons are shifted due to the shielding effect of C-1311 ring system. (D) The solution structure of the Pu22:C-1311 1:2 mol/mol non-covalent adduct.

Experiments exploiting monoacridines have clearly demonstrated that the spectra of free Pu22 and subsequent titration steps with C-1748 were practically indistinguishable (Figure 4B). In contrast, the spectra of Pu22 changed gradually upon the addition of C-1311 (Figure 4B), clearly indicating the formation of a well-defined DNA 1:2 mol/mol non-covalent adduct. Moreover, the complexation of Pu22 with C-1311 significantly increased the thermal stability of the G-quadruplex. No signs of DNA melting were visible in the NMR spectra of the Pu22:C-1311 complex recorded at 75°C, whereas the spectrum of pure Pu22 recorded at the same temperature showed evident signs of partial G-quadruplex denaturation (Figure 5C).

It should be highlighted that the addition of both UAs and C-1311 was able to induce the formation of the Pu22 G-quadruplex in solutions lacking K^+^ ions, i.e., containing unstructured DNA. Based on the integration of the resonance signals of structured and denatured Pu22 sequences, on average, ∼50% of the total DNA in a given solution was able to adopt the parallel G-quadruplex conformation upon titration with a given ligand, until an equilibrium state was reached (Figure S4 in the supplemental information). Further addition of mono- and bisacridines did not increase the G4/non-G4 ratio, but instead caused notable signal broadening, presumably due to unspecific DNA/ligand and (or) ligand/ligand interactions.

Detailed analysis of the spectra presented in Figure 4 led to the conclusion that C-1311 forms a complex with Pu22 in such a way that one ligand molecule interacts with the 5′ G-tetrad, while the other binds to the 3′ G-tetrad, as evidenced by the shifts in the imino proton resonances (Figure S5). In contrast, the interactions of C-1748 with the G-tetrads of Pu22 appear to be weak and nonspecific, as this compound induces minimal changes in the imino resonances of the studied G-quadruplex.

### NMR studies on the Pu22:C-1311 complex

Based on the observations described above, a NOESY spectrum of the Pu22 1:2 mol/mol complex was recorded (Figure 5). A fragment of the NOESY spectrum, displaying correlation signals produced by DNA aromatic protons, as well as two ligand protons, is shown below. The ligand protons are highly distinctive and can be clearly identified by their signal width (Figure 5A).

A detailed analysis of the NOESY spectrum of the Pu22:C-1311 complex revealed several key intermolecular NOE contacts (Figure 5B). These dipolar couplings confirmed that the two ligand molecules are positioned in close proximity to both external G-tetrads of the studied G-quadruplex. The C-1311 ligands exhibited NOEs to the protons of both the G7-G11-G16-G20 (5′) and G9-G13-G18-G22 (3′) tetrads. A complete list of the observed intermolecular contacts is provided in Table 1. The labeling of C-1311 protons is shown in Figure 3B, and the resonance assignments for the protons of the Pu22:C-1311 complex are listed in Table S2.

**Table 1:** Pu22:C-1311 intermolecular NOE correlations

Ligand proton	Pu22 proton	Correlation intensity
LH3	G7H8	weak
LH3	G13H8	weak
LH4	G9H1′	weak
LH10	G5H8	weak
LH10	A6H8	weak
LH18	G9H8	very weak
LH18	G17H1′	medium
LH18	G18H8	very weak
LH18	T23H1′	medium
LH18	T23H6	medium

For the explanation, how the correlation intensities were translated into distance restraints during MD simulation, please consult Table S3. For graphical presentation of selected NOEs, please consult Figure S11.

Based on the spectral data, a molecular model of the resulting complex was constructed and subjected to restrained molecular dynamics (MD) simulations. The distance restraints applied to the system were derived from the observed intermolecular NOEs (Tables 1 and S3). These calculations yielded the solution stereostructure of the Pu22:C-1311 1:2 mol/mol non-covalent adduct, representing the centroid of the dominant conformational cluster obtained from the MD trajectory (Figure 5D). The results revealed that C-1311 binds to Pu22 by occupying the “drug-binding pockets” of the G-quadruplex, intercalating between the guanine tetrads and the flanking DNA sidechains. Further details on the MD simulations and the resulting structure can be found in the discussion section.

### Free energy computations

While the molecular model of the Pu22:C-1311 complex was derived from spectroscopic studies, intrinsic 2D NMR studies were not feasible for Pu22 complexed by the unsymmetrical bisacridines (UAs) C-2045 and C-2053. Although the formation of the Pu22:UA complexes was confirmed via 1D NMR studies (Figure 4A), despite numerous attempts, no intermolecular DNA-ligand NOE contacts were detected for the Pu22:C-2045 and Pu22:C-2053 1:2 mol/mol systems. To infer the most plausible structure of a Pu22:UA adduct, one could exploit the fact that both UAs are essentially composed of a C-1311 chromophore and a C-1748 chromophore, which are connected by an aminoalkyl linker (Figure 1). Based on the observation that the imidazoacridinone C-1311 forms a well-defined, non-covalent complex with Pu22, while the 9-amino-1-nitroacridine C-1748 exhibits no specific interactions with either the 3′ or 5′ G-tetrads (Figure 4B), it is reasonable to assume that the unsymmetrical bisacridines primarily interact with the guanine tetrads of Pu22 via the imidazoacridinone (C-1311-like) ring system (Figure 1). To test this hypothesis, molecular models of these two complexes were constructed. The resulting models were subjected to MD studies, utilizing simulations with the Replica Exchange Umbrella Sampling (REUS) method. The reaction coordinate (ξ), along which the free energy profiles of the Pu22/UAs interactions were calculated, was defined as the distance between the imidazoacridinone ring system of both UAs, C-2045 and C-2053, and the external guanine tetrads of Pu22, i.e., G7-G11-G16-G20 (denoted as the 5′ G-tetrad), and G9-G13-G18-G22 (denoted as the 3′ G-tetrad).

**Figure 6:**
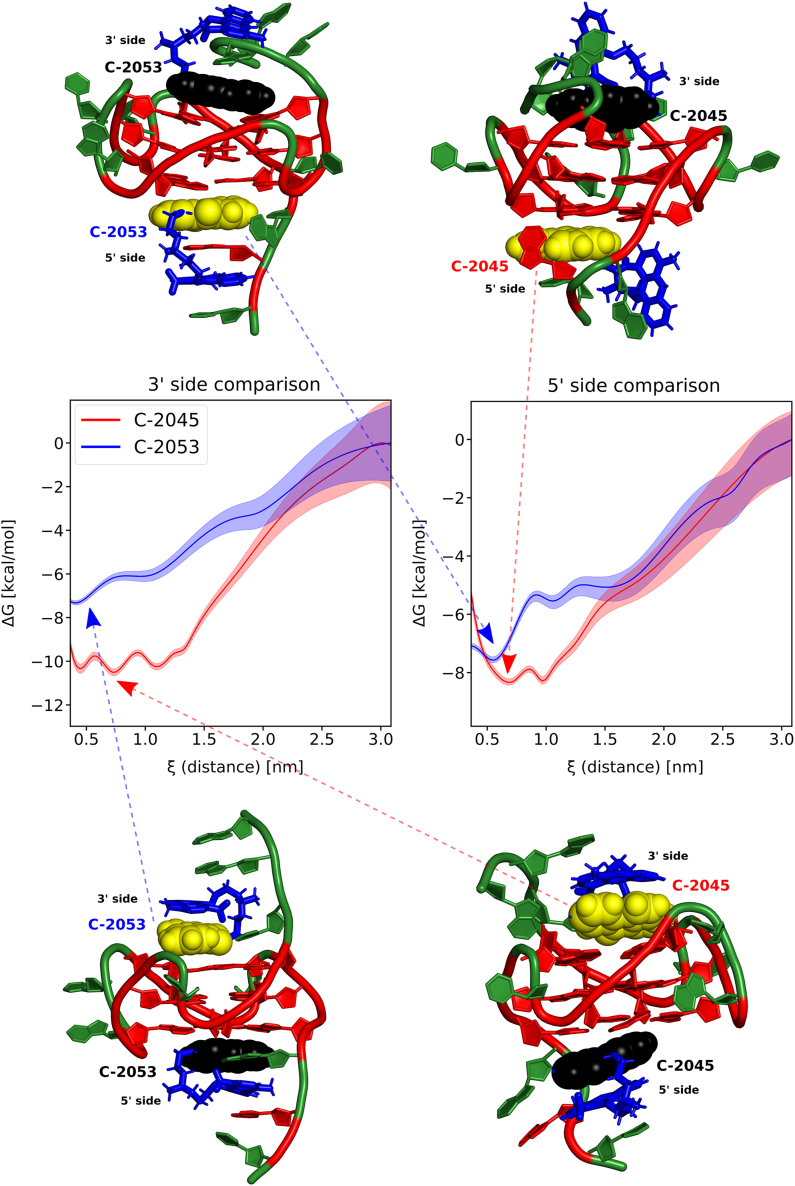
Free energy profiles of the Pu22:UA interactions, determined for the 3′ and 5′ G-tetrad, along with the representative structures corresponding to the respective minima Guanosines are depicted in red, while cytosines, thymines, and adenosines are marked in green. An aminoalkyl linker together with the 9-amino-1-nitroacridine ring is presented in blue. The imidazoacridinone moiety associated with the presented energy minimum is marked in yellow, while the other is depicted in black.

Based on 27-μs-long umbrella sampling (US) simulations, free energy profiles depicting the interactions between the 3′ and 5′ G-tetrads of Pu22 and the C-2045 and C-2053 molecules were generated. For each complex, free energy profiles were determined separately for the 3′ and 5′ G-tetrads. This means that the reaction coordinate, i.e., the distance between an external G-tetrad and the imidazoacridinone ring system of an unsymmetrical bisacridine, was sampled independently for the 5′ G-tetrad and 3′ G-tetrad during two separate simulations for each ligand, yielding four US simulations in total. A comparison of the resulting free energy profiles is presented below (Figure 6). In the case of both bisacridine ligands, the energetic minima were notably narrower at the 5′ side, indicating that the interactions between the UAs and the 5′ G-tetrad are slightly better defined than those for the 3′ G-tetrad. The profiles also suggest that C-2045 generally binds more strongly to the Pu22 G-quadruplex, whereas C-2053 establishes closer interactions. A detailed discussion of the possible sources of these disparities between the two UAs is provided in the following section of the manuscript.

Figure 6 also presents representative structures obtained via clustering of the US trajectories. Although the MD-US simulations did not employ any DNA/UAs distance restraints derived from intermolecular DNA/UAs dipolar couplings—which, as noted earlier, were undetectable in the recorded NOESY spectra—the resulting structures should be considered as models of the interactions between Pu22 and the unsymmetrical bisacridines. Nevertheless, these models, unbiased by spectral data, are in full agreement with the NMR studies of Pu22:C-1311 and Pu22:C-1748 (Figure 4B), confirming that C-2045 and C-2053 interact with Pu22 via the imidazoacridinone ring system, while the linker and 9-amino-1-nitroacridine moiety act as the ligands' sidechain (Figure 6).

## Discussion

### Monoacridine derivatives (C-1311 and C-1748)

#### C-1311

We have demonstrated that, upon titration of Pu22 with a concentrated Symadex solution, a well-defined DNA:C-1311 complex is formed, exhibiting a final 1:2 mol/mol stoichiometry. C-1311 interacts with the Pu22 G-quadruplex via its imidazoacridinone pharmacophore, with two molecules binding to the external G-tetrads: G7, G11, G16, and G20 (the 5′-side of Pu22) and G9, G13, G18, and G22 (the 3′-side of Pu22). NOE contacts involving the aromatic protons of C-1311 allowed us to localize the Symadex molecules within the so-called “drug-binding pockets,” embedded between the external G-tetrads and the flanking nitrogen bases of the 5′ (T4-G5-A6) and 3′ (T23-A24-A25) sidechains (Figure S6). Additionally, a distinctive set of intermolecular dipolar couplings between the protons of the DNA and the aminoalkyl sidechain of the ligand was recorded (Table 1). These NOEs revealed that the Symadex sidechain is well-oriented within the complex and does not exhibit significant conformational freedom. Further analysis of the Pu22:C-1311 structure revealed the formation of host/ligand hydrogen bonds within the binding cavities, involving four functional groups of the ligand serving as hydrogen bond donors, specifically the 8-OH group and the three protonated nitrogen atoms (Figures 3B, S7, and S8). Additionally, the N5 and O6 atoms of the ligand may also serve as hydrogen bond acceptors (Figure 3B).

Another factor determining the resulting structure of the Pu22:C-1311 complex is that one of the nitrogen atoms in the imidazole-type ring of C-1311 (N2) becomes protonated below pH ∼8, thereby carrying a positive charge (Figure 3B). Apart from hydrogen bonds formed by this group, molecular modeling studies revealed that the N2 nitrogen atom is coordinated by the O6 atoms of guanine residues forming the external G-tetrads, similar to the coordination of K^+^ cations trapped between the G-tetrad planes. Consequently, a column of four charged atoms is formed along the central channel of the G-quadruplex, consisting of two N^+^ atoms from the two C-1311 molecules and two K^+^ cations (Figure S9). Analogous conjugated systems have been previously observed in G4/ligand complexes, particularly in studies of alkaloids,^45^ including berberine.^46^ Berberine molecules bind at both the 5′ and 3′ ends of the G-tetrads, forming a 1:2 G-quadruplex/ligand complex. On both sides of the complex, the positively charged convex side of the molecule points toward the center of each G-tetrad.^46^ In another study, the structure of the fluorinated acridine RHPS4, known as a potent telomerase inhibitor, was solved by NMR in complex with the parallel tetramolecular G-quadruplex h-tel7. A 1:2 G-quadruplex/ligand stoichiometry was observed, with two RHPS4 molecules stacked onto both outer G-tetrads. Interestingly, at the 5′ end, the acridine moiety intercalated between the G-tetrad and the A-tetrad, further stabilizing the overall structure. Furthermore, the partial positive charge at position 13-N of the acridine ring acted as a “pseudo-potassium” cation, aligning with the central channel of the G-quadruplex.^47^

Notably, not all acridine ligands interact with both external G-tetrads of G-quadruplexes. For instance, phenylaminoacridine, also known as BRACO19, considered a benchmark G4 binder, can be used as a fluorescent tag in exchange experiments to confirm G4 binding by competing ligands. BRACO19 stacks directly onto the 3′ G-tetrad face of the bimolecular human telomeric G-quadruplex, as shown by X-ray studies.^48^ Another acridine derivative, BSU6039, is sandwiched between two guanine residues of the 5′ G-tetrad and one thymidine residue from the TTTT loops of the *Oxytricha nova* telomeric DNA sequence, with the complex stabilized by stacking interactions.^49^ Furthermore, additional 3,6-disubstituted acridine derivatives have been evaluated, all exhibiting similar binding modes to BSU6039.^50^ As a side note, another 9-aminoacridine derivative (without a specific name) was even able to switch the G-quadruplex topology from antiparallel to parallel in the promoter region of Nrf2 (nuclear factor erythroid 2-related factor 2), although the exact stoichiometry and structure of this complex remain unknown.^51^

The next aspect worth discussing is the presence and role of the ligand’s sidechain in G-quadruplex/ligand interactions. C-1311 contains a positively charged nitrogen atom in its sidechain, significantly increasing the molecule’s DNA-binding affinity. This sidechain is also responsible for forming contemporary hydrogen bonds with the sugar-phosphate backbone of the flanking residues of Pu22. In general, the role of the ligand’s sidechain in G-quadruplex/ligand interactions is crucial, both in terms of binding strength and ligand preference for specific G4 topologies. The literature contains numerous reports on this topic. For instance, Ji and coworkers examined the interaction of various natural alkaloids with G-quadruplexes formed by *c-MYC* Pu27. Among them, sanguinarine, berberine, palmatine, and tetrahydropalmatine can induce the formation and stabilization of G-quadruplexes.^45^ Notably, a 9-substituted berberine derivative with an alkyl sidechain carrying a terminal amino group, synthesized by Huang and colleagues, exhibited higher binding affinity to G-quadruplexes than the parent compound.^52^^,^^53^ Pivetta and colleagues synthesized several perylene derivatives with linear or cyclic amines in the sidechains, showing that the binding affinity and selectivity of perylenes for G-quadruplexes depend on the sidechain structure.^54^ A certain indoloquinoline derivative can distinguish between different G4 topologies, showing a preference for parallel-stranded MYC quadruplexes but also interacting with hybrid forms. Its crescent-shaped, non-coplanar structure has limited stacking interactions, but the sidechain contributes to better discrimination between different G4 topologies.^55^ In a theoretical study, Arba et al. explored the binding of porphyrin derivatives to different G4 topologies, concluding that G-quadruplex binding favors ligands with more substituents due to favorable electrostatic interactions between the positive charge of the substituent/sidechain and the negative charge of the phosphate backbone.^56^ These reports lead to the conclusion that the structure of the sidechain is the primary factor determining the selectivity and efficacy of a ligand’s binding to a given G-quadruplex. This conclusion aligns with our previous findings, which showed that the sidechain structure of monoacridines interacting with double-stranded DNA substantially impacts the dissociation constant between the DNA host and the ligand guest.^57^^,^^58^

#### C-1748

Although some changes in the chemical shift (Δδ) of the imino DNA protons were observed during the titration of Pu22 with nitroacridine C-1748, these shifts were barely detectable and limited to only two imino signals, originating from the G-tetrad located at the 5′ end of the G-quadruplex. The resulting imino resonance pattern was notably different when compared to other DNA:ligand systems discussed in this study (Figure 4). This suggests that C-1748 does not exhibit significant affinity for the surfaces of the 5′ and 3′ G-tetrads of Pu22. Even if such interactions do occur, they are likely rapid, unstable, and transient, making them difficult to detect or characterize through NMR techniques. While this outcome may seem counterintuitive, it aligns with our previous studies on the interactions of monomeric acridines with DNA duplexes.^57^^,^^58^ Imidazoacridinone C-1311, for example, was identified as a potent dsDNA intercalator with high affinity for TA/TA dinucleotide steps, whereas nitroacridines such as C-283 and C-1748 displayed no measurable intercalative potential.^58^ This observation is likely linked to the non-planar geometry of 9-amino-1-nitroacridine molecules.^58^^,^^59^ The characteristic butterfly-shaped stereostructure of C-1748 may therefore once again explain the lack of strong π-π interactions between this ligand and the guanine bases of Pu22. Nevertheless, noticeable Δδs of the thymidine H6 protons were recorded, indicating that C-1748 may engage in nonspecific interactions with the loops of the G-quadruplex or with its 5′ and/or 3′ flanking regions. However, due to the highly dynamic nature of the studied system, the exact nature of these interactions could not be definitively characterized.

While this mode of Pu22/C-1748 interaction is unexpected, it is not unprecedented. Recent NMR studies have elucidated the structures of two complexes between the parallel tetramolecular G-quadruplex h-tel7, composed of the sequence d(TTAGGGT), and two anthracyclines: epirubicin^30^ and doxorubicin (*syn.* adriamycin).^31^ Despite being well-known dsDNA intercalators, these compounds do not bind at the external G-tetrads of the G-quadruplex, instead localizing within the grooves, where they can adopt two different orientations. Conversely, the structure of another anthracycline, daunomycin, in complex with the tetramolecular parallel G-quadruplex *o*-tel6, composed of the sequence d(TGGGGT), was solved by X-ray crystallography.^60^ In this case, three daunomycin molecules, held together in one layer by a network of van der Waals contacts, interacted with the 5′ G-tetrad via stacking interactions. Given that these three anthracyclines are structurally very similar, yet exhibit entirely different binding modes to G4 structures, an additional issue arises: the influence of the experimental technique used, particularly the phenomenon of crystal packing. This is a topic for a separate discussion, which has already been addressed in the literature.^18^

### Unsymmetrical bisacridines (C-2045 and C-2053)

The complexes formed between Pu22 and the unsymmetrical bisacridines (UAs) C-2045 and C-2053 were, as in the case of C-1311, clearly evidenced by gradual shifts in the imino proton resonances of the G-quadruplex, observed during the stepwise addition of the respective ligand (Figure 4). While one might argue that the final spectra were not as sharp as those recorded for the Pu22:C-1311 system, the resulting complexes were unambiguously determined as DNA:UAs with a 1:2 mol/mol stoichiometries. Unfortunately, the NOESY spectra of the Pu22:UAs complexes did not display any correlations involving the aromatic protons of the UAs, or the correlations that were detected were extremely weak, intramolecular, and derived solely from the imidazoacridinone subunit. Consequently, the registration of intermolecular DNA/ligand NOEs was not feasible. This implies that the ligand resonances were significantly broadened, indicating that the chemical environment of the UAs was not stable or well-defined within the complex, further suggesting the absence of a single dominant Pu22:UAs structure in the studied solution.

This peculiar stereostructural polymorphism of the Pu22:UAs complexes can be attributed to the dimeric nature of the UAs, which likely accounts for the differences between the imino proton patterns in the spectra of Pu22:C-1311 and Pu22:UAs systems. Given that UAs consist of two monoacridine subunits—one being the imidazoacridinone pharmacophore and the other the 9-amino-1-nitroacridine moiety—and considering the previously described differences in the interactions of Symadex (imidazoacridinone) and C-1748 (9-amino-1-nitroacridine) with the studied G-quadruplex, it can be inferred that the binding of UAs to the 5′- and 3′-G-tetrads of Pu22 likely occurs via the imidazoacridinone ring system. The aminoalkyl linker and the 9-amino-1-nitroacridine moiety likely function as a large sidechain of the dimeric ligand. This interpretation suggests a wide range of potential interactions between the UA ligand sidechain and the 5′ and 3′ flanking residues of the G-quadruplex, as well as its loops. Although these interactions could not be analyzed by NMR, computational approaches using US techniques were employed.

The computational results for the Pu22:C-2045 system revealed the presence of deep and wide energetic minima for both ligand molecules interacting with the external G-tetrads of the G-quadruplex. Since the reaction coordinate (ξ) was defined as the distance between the imidazoacridinone ring system and the guanine tetrads, minima ranging from 0.5 to 1.0 nm indicate that DNA/ligand binding is not solely driven by π-π interactions between the imidazoacridinone subunit and a G-tetrad, but also involves the entanglement of the entire C-2045 molecule with one of the Pu22 sidechains. The energetic minimum is slightly broader on the 3′ side of the G-quadruplex, extending to ∼1.3 nm, which is likely due to the different sequences of the sidechains (TGA at the 5′ end vs. TAA at the 3′ end). The recorded molecular dynamics (MD) trajectories indicated that the imidazoacridinone pharmacophore interacts with all the nucleotides of the 5′ sidechain and simultaneously blocks the 9-amino-1-nitroacridine moiety on the surface of the 5′ G-tetrad, a behavior not observed on the 3′ side. Conversely, when the imidazoacridinone ring system interacts with the G-tetrad plane, which corresponds to the energetic minima at ∼0.7 nm, the remaining 9-amino-1-nitroacridine moiety engages with the flanking residues, as shown in Figure 6.

The results for the Pu22:C-2053 system were markedly different from those for C-2045. The free energy profiles exhibited a single, relatively sharp global minimum at ξ ∼0.5 nm, corresponding to π-π interactions between the imidazoacridinone moiety and the external G-tetrads. Distances between ξ ∼0.5 and ∼1.0 nm were less favorable, with the largest difference observed on the 5′ side of the complex (∼2.5 kcal/mol). The structural difference between C-2045 and C-2053 lies in the presence of a hydroxyl group at position 8 of the imidazoacridinone ring system (Figure 1). In studies of the intercalative binding of monoacridines to DNA duplexes, this hydroxyl group was found to be crucial, primarily due to the formation of hydrogen bonds between the 8-OH and the 4′-oxygen atom of 3′-adjacent deoxyribose moiety.^57^^,^^58^ Similarly, in the binding of acridines to Pu22, this hydroxyl group appears to play an important role. The presence of the 8-OH group not only alters the electronic properties and polarity of the imidazoacridinone ring system, but also facilitates the formation of hydrogen bonds with the G-quadruplex, including its sidechains. Consequently, while C-2045 generally binds more strongly to Pu22 (ΔΔG ∼2–3 kcal/mol) through π-electrons, van der Waals interactions, and hydrogen bonds, it does not adopt a single dominant structure due to its numerous conformational possibilities, leading to broader energetic minima. In contrast, C-2053, lacking the 8-OH group, favors π-electronic interactions between its imidazoacridinone subunit and the G-tetrads, but its overall binding to Pu22 is slightly weaker (Figure S10). Both scenarios result in broadened imino proton resonances of Pu22, as observed in the NMR spectra.

**Figure 7:**
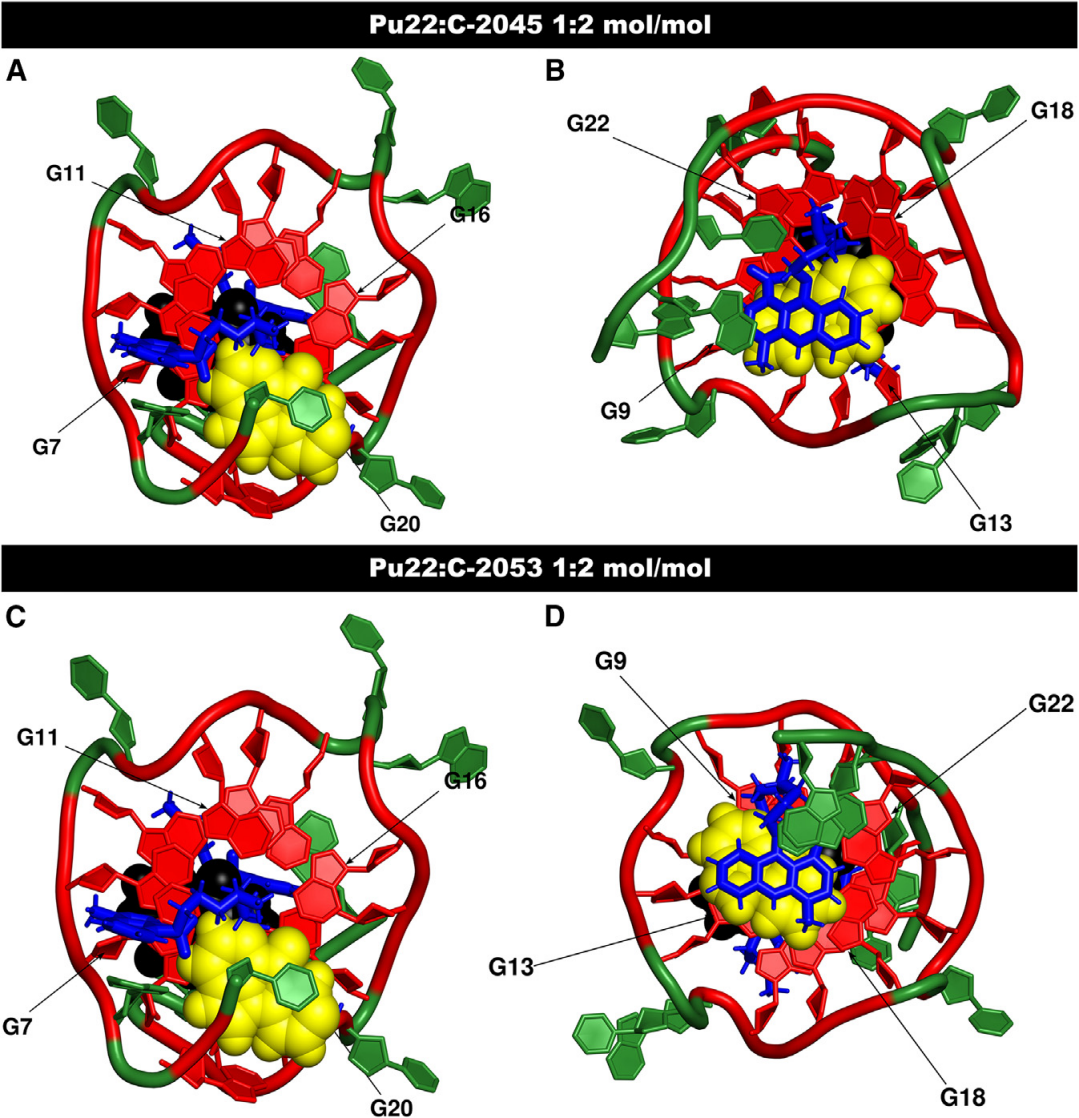
Axial depictions of the Pu22:UAs complexes (A) Pu22:C-2045, 5′ side. (B) Pu22:C-2045, 3′ side. (C) Pu22:C-2053, 5′ side. (D) Pu22:C-2053, 3′ side. Imidazoacridinone ring systems are depicted in yellow, 9-amino-1-nitroacridine ring systems and the UAs’ aminoalkyl linkers are depicted in blue, G-tetrads are depicted in red, non-G nucleotides are depicted in green.

In conclusion, the MD-US trajectories for both Pu22:UAs complexes revealed several interesting structures, examples of which are shown in Figures 6 and 7. In these structures, both ring systems of the unsymmetrical bisacridines participate in π-electron interactions, intercalation between the G-tetrads and flanking residues (‘drug-binding pockets’), intercalation between the nitrogenous base residues of the flanking regions, short-lived base pairing interactions reminiscent of Watson-Crick base pairing, and hydrogen bond formation (primarily C-2045). These hydrogen bonding interactions likely contribute to the broader energetic minima observed for C-2045, as this ligand has more extensive interactions with the G-quadruplex sidechains, as well as they may explain its slightly stronger overall binding to Pu22 while compared to C-2053. It is also worth noting that the imidazoacridinone ring systems of the unsymmetrical bisacridines are positively charged, similar to C-1311 (Figure 3), both in the conditions used for NMR spectroscopy and in the computational experiments. As discussed earlier, in the Pu22:C-1311 complex with a 1:2 mol/mol stoichiometry, the positively charged nitrogen atoms of the ligand aligned with the K^+^ cations trapped between the G-tetrad planes. However, in the computational experiments with Pu22:C-2045 and Pu22:C-2053 complexes, such an arrangement of the positively charged nitrogen atoms of the imidazoacridinone subunits was not observed. This result can be understood by considering the aminoalkyl linker and the 9-amino-1-nitroacridine moiety of the UAs as effectively functioning as an extended “sidechain” of the imidazoacridinone ring system. From this perspective, the UAs’ sidechain is substantially larger compared with that of C-1311, offering more opportunities for DNA/ligand interactions, which diminishes the role of the imidazoacridinone’s positive charge in determining the structure of the complex. Nevertheless, this charge remains important, as it significantly enhances the overall affinity of UAs for G-quadruplex structures.

It is also worth mentioning that this is not the first study to explore the interactions between bisacridines and G-quadruplex structures. In 2020, Kuang et al. synthesized symmetrical bisacridine derivatives differing in the length of their aminoalkyl linkers. Their research strategy aimed to optimize bisacridine structures for their efficacy in simultaneously targeting the G-quadruplex in the *c-MYC* oncogene promoter and the associated i-motif DNA structure. The results showed that bisacridines with longer aminoalkyl linkers provided the greatest thermal stabilization of both the G4 and i-motif structures.^61^ Unfortunately, these studies were not accompanied by detailed structural analyses, partly because, so far, the structure of the *c-MYC* promoter i-motif has not been elucidated by NMR or X-ray crystallography.^61^^,^^62^^,^^63^ Therefore, detailed structural studies on the interactions of unsymmetrical bisacridines with i-motifs, particularly the one from the *c-MYC* promoter, represent an exciting avenue for future research.

### Conclusions

Based on the experimental data collected, we have demonstrated that the monoacridine derivative C-1311 (Symadex), as well as the unsymmetrical bisacridines (UAs), codenamed C-2045 and C-2053, indeed interact with the Pu22 G-quadruplex, a mutated sequence derived from the promoter region of the *c-MYC* protooncogene. These interactions were modeled using both spectroscopic and computational techniques. In contrast, the 9-amino-1-nitroacridine derivative C-1748, a direct descendant of the first Polish anticancer drug, Nitracrine/Ledakrin (C-283), did not exhibit strong or well-defined interactions with the studied sequence. This result aligns with the general observation that the presence of an extended aromatic residue is not sufficient to ensure stable binding on the upper or lower G-tetrads of the G4 structure. As noted in a recent review by Biver, "the inspection of the literature enlightens a picture where each system makes its own story, as each of the molecules has a reactivity that is the sum of subtle details that are hard to pre-evaluate."^64^

Taking into consideration all the findings discussed above, it should be concluded that the monoacridine C-1311, along with the bisacridines C-2045 and C-2053, exhibit notable affinity for the Pu22 G-quadruplex. However, the nature of these interactions is strongly dependent on the structure of the ligand’s sidechain and whether the interaction occurs on the 5′ or 3′ end of Pu22. The complexity of DNA/ligand interactions increases with the size of the ligand’s sidechain, as reflected in both NMR experiments (broadened resonance signals) and computational studies (broadened energetic minima). On the other hand, the interactions between Pu22 and C-1311, as well as between Pu22 and UAs, are generally better defined at the 5′ end of the G-quadruplex, which contains the 5′-TGA-3′ flanking residues. Nevertheless, it must be acknowledged that in a cellular environment, a DNA G-quadruplex is only one part of a much larger molecular structure, and the three-nucleotide-long sidechains of the G4 sequence discussed in this study do not fully reflect the real-life scenario. The true G-quadruplex sidechains are much longer and presumably less prone to reorientation, which represents a major limitation of our study.

The imidazoacridinone pharmacophore has been demonstrated to be a good example of a planar molecule capable of interacting with the MYC-G4 structure and is a strong candidate as a small-molecule agent capable of suppressing *c-MYC* expression. In light of the fact that imidazoacridine C-1311 is also a potent intercalator of double-stranded DNA (dsDNA), while the unsymmetrical acridines do not exhibit significant interactions with dsDNA,^58^ UAs have a significant advantage as potential drugs aimed at the downregulation of *c-MYC* expression. While strong interactions with both dsDNA^58^ and G4 structures tend to disqualify Symadex as a selective ligand for G-quadruplexes, this does not exclude its binding to G-quadruplexes as a potentially important aspect of its molecular mechanism of biological activity, which is still under investigation.

Recently, the cytotoxicity of four unsymmetrical bisacridines, including C-2045 and C-2053, was evaluated against colorectal cancer cells (HCT116) and lung cancer cells (H460), in comparison with “normal” cell lines CCD 841 CoN and MRC-5.^5^ Using RT-PCR and western blotting techniques, it was demonstrated that cancer cells exhibit greater sensitivity to the presence of UAs compared with healthy cells, though the response varies between cell lines.

In HCT116 colorectal cancer cells, *MYC* expression was found to increase, while c-Myc protein translation occurred at a level comparable to that in healthy cells. In contrast, for H460 lung cancer cells, *MYC* transcription was similar to that in normal lung cells; however, the c-Myc protein level was reduced nearly to undetectable levels. Given that G-quadruplex structures are also present in RNA, these results may suggest that the stabilization of G-quadruplexes by unsymmetrical bisacridines occurs within mRNA rather than the DNA, leading to unchanged (or increased) transcription while simultaneously inhibiting c-Myc protein translation.

In another study, western blotting experiments demonstrated that UAs significantly suppress c-Myc protein expression in several pancreatic cancer cell lines, including those with both wild-type and mutated NHE regions, which harbor different variants of the Pu27 G-quadruplex.^65^ In some cases, after 72 h of incubation, c-Myc protein levels were barely detectable. The mechanism underlying this suppression may be analogous to that observed in the previously mentioned H460 lung cancer cell line. Furthermore, cell viability assays confirmed that all tested unsymmetrical bisacridines effectively inhibited the proliferation of all pancreatic cancer cell lines examined, while exhibiting lower toxicity toward healthy cells compared with the positive control, gemcitabine.

It is noteworthy that markedly different results were obtained within the same cell lines when examining the effect of UAs on *K-RAS* expression, which contains a parallel G-quadruplex-forming sequence in its promoter region, known as 22RT.^66^ In preliminary studies, we demonstrated that both C-1311 and UAs interact with the 22RT G-quadruplex; however, their binding modes differ from those observed with the Pu22 sequence. Similar findings have emerged from initial studies on the interaction of imidazoacridinones with the telomeric h-tel22 sequence (L.T. and P.J., unpublished results), which also forms a G-quadruplex but adopts a different topology.^64^

Thus, our future research will focus on the role of imidazoacridinone side chains in binding to various G-quadruplexes, with particular attention to the influence of side chain structure, size, and mobility.

## Materials and methods

### Chemicals

5-Diethylaminoethylamino-8-hydroxyimidazoacridinone (C-1311/Symadex), 9-(2′-hydroxyethylamino)-4-methyl-1-nitroacridine (C-1748), 9-{N-[(8-hydroxyimidazo[4,5,1-de]-acridin-6-on-5-yl)aminopropyl]-N-methylaminopropylamino}-4′-methyl-1′-nitroacridine×3HCl (C-2045), and 9-{N-[(imidazo[4,5,1-de]-acridin-6-on-5-yl)aminopropyl]-N-methylaminopropylamino}-4′-methyl-1′-nitroacridine×3HCl (C-2053) were synthesized at the Department of Pharmaceutical Technology and Biochemistry, Faculty of Chemistry, Gdańsk University of Technology. Pu22 DNA G-quadruplex sequence was purchased from Metabion, GmbH and additionally purified from triethylamine acetate using Amicon Ultra 2 mL centrifugal filters (3 kDa cutoff) provided by Merck, GmbH. The purification with Amicon filters involved washing of DNA material with 2 M LiCl aqueous solution, a then multiple washing with deionized H_2_O at 18°C. All compounds were >95% pure by HPLC analysis.

### Sample preparation

Acridine derivatives (C-1311, C-1748, C-2045, and C-2053) were dissolved in a deionized water (conductivity 0.056 μS cm^−1^, Milli-Q water purification system; Merck Millipore) to obtain 10 mM ligand stock solutions. These solutions were used for the titration of Pu22 samples.

Each Pu22 sample subjected to NMR studies contained 4 OD (∼90 μM) or 20 OD (∼450 μM) DNA material, dissolved in 10 mM potassium cacodylate buffer of pH 5.0, with the addition of 10 mM KCl. Total volume of a sample was 200 μL. Samples were originally prepared in H_2_O/D_2_O 9:1 v/v, then—if necessary—the solvent system was removed and 99.98% pure D_2_O was added to reach 200 μL final volume of a solution. DNA solutions of 4 OD were used for the 1D NMR studies on the formation of Pu22:ligand complexes, whereas 20 OD samples were used as an upscale designed for the 2D NMR studies.

### NMR experiments

Spectra were collected on a 700-MHz Brüker Avance III spectrometer equipped with a QCl CryoProbe, installed at the Institute of Bioorganic Chemistry, Polish Academy of Sciences, Poznań, Poland. All spectra were processed using Brüker TopSpin 3.6.5 software and NMRFAM-SPARKY v1.470 suite.^67^ Proton 1D NMR spectra were collected using standard parameters, at temperatures ranging from 5°C to 75°C. Reference NMR spectra for Pu22 consisted of NOESY (150 ms mixing time) and TOCSY (60 ms spin-lock time) experiments, conducted at 50°C in H_2_O/D_2_O 9:1 v/v solvent system, as well as NOESY (150 and 400 ms mixing time), TOCSY (60 ms spin-lock time), and HC-HSQC spectra acquired in 100% D_2_O at 50°C. The resonance assignment process itself was performed using standard approaches.^68^ For the assignment of the dsDNA:ligand complexes and for the identification of the DNA/ligand cross-peaks, NOESY (400 ms mixing time) and TOCSY (60 ms spin-lock time) spectra were recorded for the complexes at 50°C in H_2_O/D_2_O 9:1 v/v solvent system.

### Molecular modeling

#### Quantum chemical parametrization

The numerical models of C-1311, C-2045, and C-2053 were built using the Avogadro software. Initial geometries were optimized using the MMFF94 force field. Then, basic parameters for both ligands were taken from the latest version of CHARMM36 Generalized Force Field,^69^ using a computational tool available at paramchem.org. Subsequently, the models were refined using Gaussian09 software.^70^ Optimal geometries were found with Minnesota hybrid functionals (MN12SX) at the 6-31G∗ level of theory. Then, partial atomic charges were computed using a second-order Møller-Plesset perturbation theory (MP2). Models prepared in this way were later used in all the simulations.

#### Preparation of the simulation systems

Numerical model of G-quadruplex was taken from Protein DataBank (PDB), model number PDB: 2L7V.^20^ Based on the results of the 1D NMR examinations, in each of the three simulation systems, two ligand molecules (C-1311, C-2045, or C-2053) were placed near the Pu22 G-quadruplex in a way that the imidazoacridinone ring systems were located in proximity of the external G-tetrads: one near the 3′ side, whereas the second next to the 5′ guanine tetrad. These initial models of the complexes were built using VMD v1.9.4. software.^71^

#### Simulation procedures

Resulting Pu22:ligand complexes were placed in cubic boxes with the diameter of 6.2 nm. Subsequently, all built systems were minimized in a vacuum to find the initial minimum of potential energy function. The simulation boxes were filled with water (spc216 model). The energy of a system was again minimized and, in the next step, the environment was neutralized by adding an appropriate amount of potassium and chlorine ions to reach 10-mM concentration. Two potassium ions were kept between the G-tetrads of Pu22 during all computations. As a final step of preparation, the energies were minimized once again and all three systems were subjected to 50-ns-long MD simulations with position restraints applied to non-hydrogen atoms of DNA and ligands. For the MD experiments, the CHARMM36 force field^69^ and the GROMACS 2020.4 package^72^ equipped with the PLUMED 2.6.2 plug-in were used. To maintain the continuity of the simulated system, periodic boundary conditions were applied in all three dimensions. From a statistical thermodynamic point of view, all simulations were performed in the NPT ensemble, i.e., maintaining constant pressure and temperature. The temperature of the systems during the simulation was kept at 323 K (to match the temperature of the 2D NMR experiments) using a Nosé-Hoover thermostat, while the pressure was kept at 1 bar using the Parrinello-Rahman barostat. The equations of motion were integrated using Verlet’s algorithm with a time step of 2 fs.

#### Restrained MD simulation of Pu22:C-1311 complex

After the Pu22:C-1311 system was prepared and equilibrated, it was subjected to 1,000 ns restrained molecular dynamics simulation. During this run, distance restraints corresponding to the NOE contacts between the protons of the ligand and the protons of the DNA were applied (Table 1). This was done using the GROMACS implementation of the restraining potential, which adds a quadratic penalty to the potential when an interatomic distance exceeds a lower or upper threshold (see Table S3 in the supplemental information). The same force constants of 239 kcal mol^−1^·nm^−2^ were used for all restrained distances, corresponding to the DNA/ligand intermolecular contacts. Cluster analysis of the resulting trajectory was performed using the Daura method^73^ with an RMSD cutoff set to 0.25 nm. The center structure of a dominant conformational cluster (67.5% of the simulation time) was extracted and presented as the most representative structure of Pu22:C-1311 non-covalent adduct (Figure 5).

#### US of Pu22 complexed by UAs

The Hamiltonian REUS method was employed to obtain free energy profiles of DNA:UA interactions.^74^^,^^75^ The reaction coordinate, ξ, was defined as the distance between the center of mass determined for the 3′ and 5′ Pu22 G-tetrads and the center of mass of the imidazoacridinone ring system of a bisacridine ligand, located near the respective guanine tetrad. In order to ensure the stability of Pu22 G-quadruplex structure, distance restraints between two K^+^ ions embedded between G-tetrads and the O6 oxygen atoms of guanine residues, forming corresponding G4 planes and coordinating the potassium cations were introduced and kept during all the US simulations.

To avoid bias in starting configuration for US simulations, the following methodology was employed. Initially, in the case of each system, two ligand molecules were placed in a moderate proximity to 3′ and 5′ G-tetrad planes of Pu22. Then, simulations of the formation of G4:ligand complexes were conducted incorporating distance restraints, derived from Nuclear Overhauser Effect Spectroscopy (NOESY) data for Pu22:C-1311 complex. This resulted in formation of two Pu22:UAs complexes, in which the imidazoacridinone moieties of C-2045 and C-2053 were attached to 3′ and 5′ G-tetrad planes, while the rest of the ligand molecules were given full conformational freedom. After this run, NMR-based distance restraints were removed. The resulting complexes were then subjected to 100-ns simulations, in which the reaction coordinate (DNA/ligand ξ distance) was increased from 0.4 to 3.0 nm by the applied harmonic potential with a force constant of 597.5 kcal mol^−1^·nm^2^. The initial structures for US calculations along the reaction coordinate were selected with 0.1-nm spacing from these trajectories, which resulted in 27 windows for all studied variants. Consequently, each window was equilibrated for 50 ns. After all those steps, every window was simulated for 1,000 ns, yielding 27 μs of total simulation time of each system. To increase conformational sampling, the REUS approach with 1,000 ps exchange rate between windows was applied.

During each run, only one ξ was studied (for instance: the distance between C-2045 and the G-tetrad at the 5′ side of Pu22), whereas at the opposite side of the G-quadruplex, a given ligand was constantly kept close to the guanine tetrad at the distance of ∼0.4 nm using additional harmonic potential. That distance was defined in the same way as the reaction coordinate, whereas the force constant of the potential was equal to 597.5 kcal mol^−1^·nm^2^. Therefore, for a given Pu22:UAs complex, each side of the G-quadruplex (3′ and 5′) was studied separately, i.e., two US simulations for Pu22:C-2045 and two for Pu22:C-2053 were conducted, resulting in four US runs in total. The weighted histogram method (WHAM) was used to obtain the free energy profiles.^76^

## Data availability

All data needed to evaluate the conclusions in the paper are present in the paper and/or the supplemental information. The raw NMR spectra and MD trajectories can be provided by the corresponding author pending scientific review and a completed material transfer agreement. Requests for the aforementioned data should be submitted to tomasz.laskowski@pg.edu.pl.

## Acknowledgments

This work was financed by the 10.13039/100025296OPUS (Poland), a grant from the Polish National Science Center no. 2019/33/B/NZ7/02534. Computational resources were provided by TASK (Gdańsk, Poland).

## Author contributions

T.L. conceived the experiments; M.K., W.A., and T.L. conducted the spectroscopic measurements; M.K., J.B.-B., and T.L. conducted and analyzed the calculations; M.K., J.P., R.S., and T.L. analyzed the spectra; E.P. synthesized the acridine derivatives; Z.M. acquired the funding and supervised the project; T.L. and M.K. wrote the initial version of the manuscript and prepared all the figures. All authors have read, revised and accepted the manuscript in its current form.

## Declaration of interests

The authors declare no competing interests.

## Declaration of generative AI and AI-assisted technologies in the writing process

During the preparation of this work the authors used ChatGPT-4o in order to improve language. After using this tool, the authors reviewed and edited the content as needed and take full responsibility for the content of the publication.

## Supplemental information

Document S1. Figures S1–S11 and Tables S1–S3

Document S2. Article plus supplemental information

## Footnotes

Supplemental information can be found online at https://doi.org/10.1016/j.omtn.2025.102513.
