# Supplementary material for: The interactions of Pu22 G-quadruplex, derived from c-MYC promoter sequence, with antitumor acridine derivatives—An NMR/MD combined study
Source: Mol Ther Nucleic Acids. 2025 Mar 13;36(2):102513. doi: 10.1016/j.omtn.2025.102513 (PMC11986977; doi:10.1016/j.omtn.2025.102513)
Supplement: Document S1. Figures S1–S11 and Tables S1–S3 [file mmc1.pdf]

OMTN, Volume *36*

Supplemental information

The interactions of Pu22 G-quadruplex, derived from *c-MYC* promoter sequence, with antitumor acridine derivatives—An NMR/MD combined study

Tomasz Laskowski, Michał Kosno, Witold Andrałojć, Julia Pakuła, Rafał Stojałowski, Julia Borzyszkowska-Bukowska, Ewa Paluszkiewicz, and Zofia Mazerska

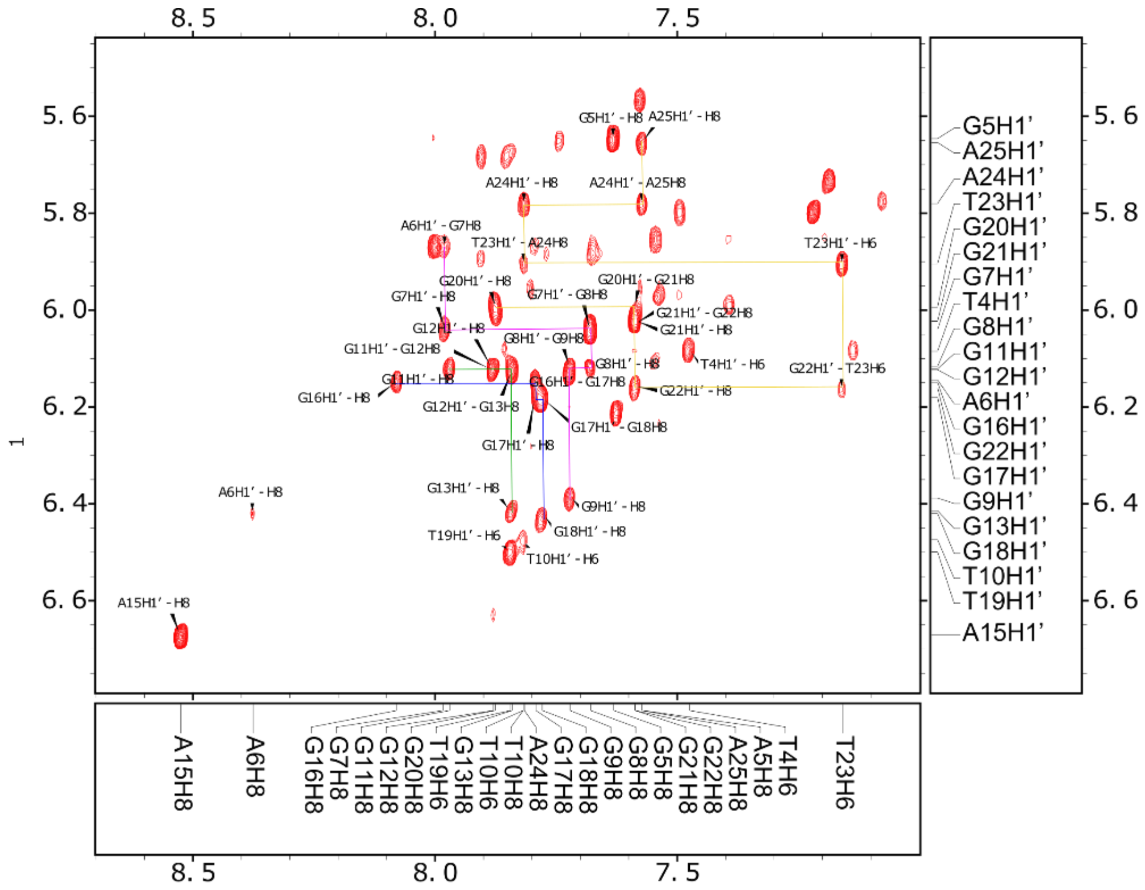

Figure S1. The expanded H8/H6-H1' region of the 2D-NOESY spectrum of the Pu22. Axes are presented in [ppm].

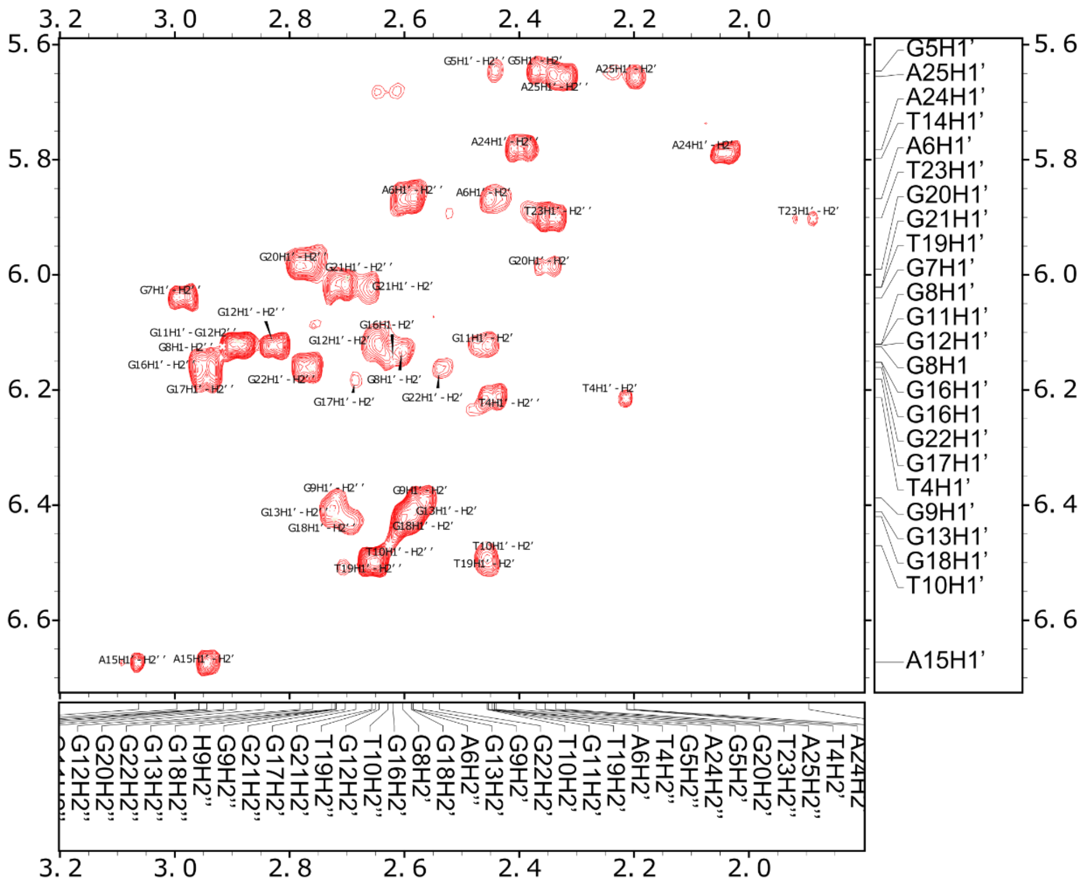

Figure S2. The expanded H1'/H2''-H2' region of the 2D-NOESY spectrum of the Pu22. Axes are presented in [ppm].

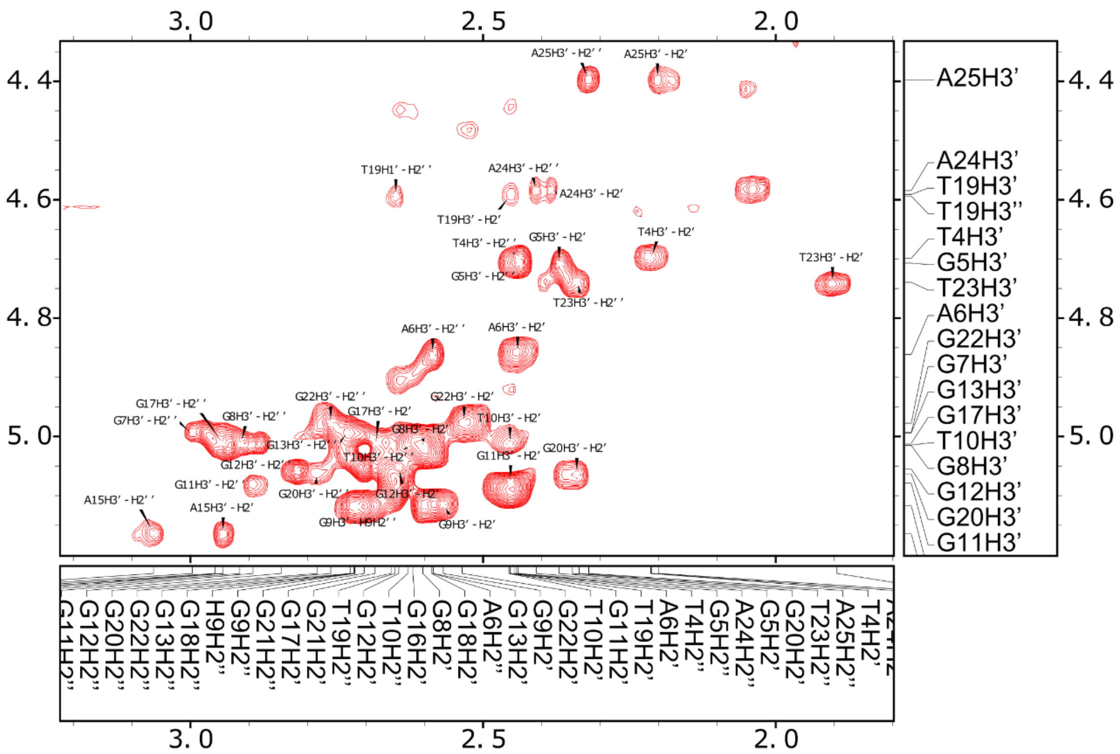

Figure S3. The expanded H2''-H2'/H3' region of the 2D-NOESY spectrum of the Pu22. Axes are presented in [ppm].

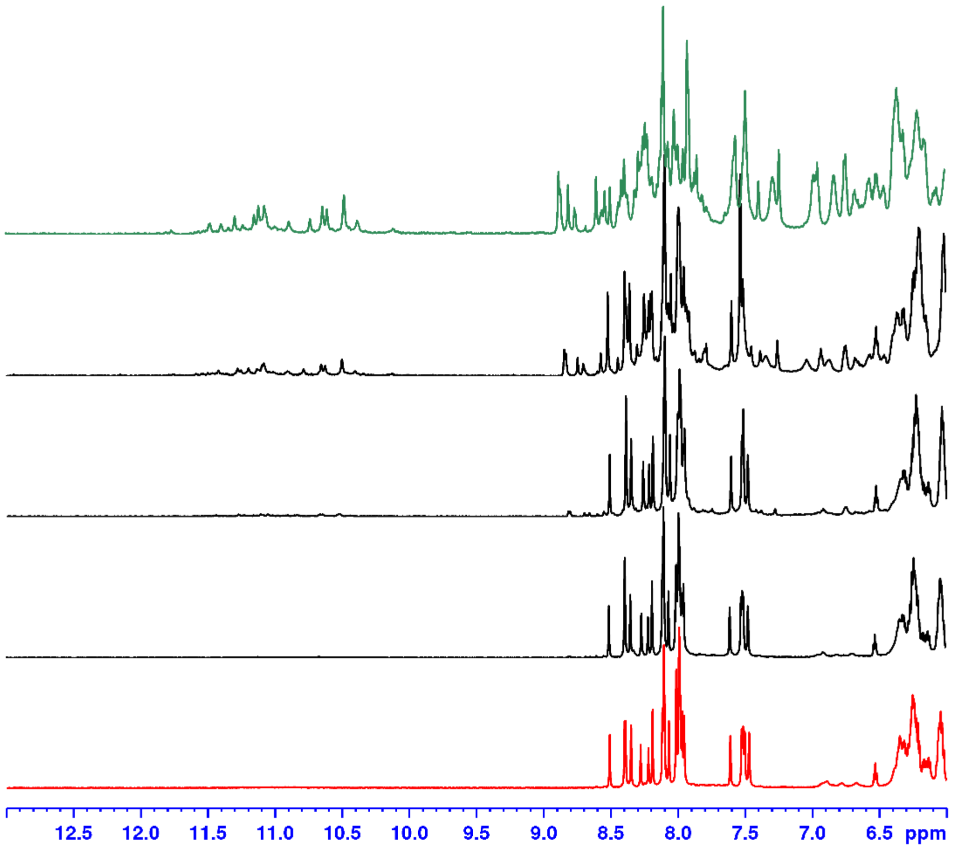

Figure S4. Titration of unstructured Pu22 sequence by C-1311 solution. Red spectrum: free DNA, each consecutive spectrum represents the addition of 0.5 molar equivalent of the ligand (in relation to Pu22). Green spectrum: the equilibrium state between unstructured DNA and Pu22 G-quadruplex, folding upon the presence of the ligand. Spectra recorded at 25 °C in deionized H_2O/D_2O 9:1 mol/mol solvent system, without the addition of any $Na^+/K^+/NH_4^+$ ions.

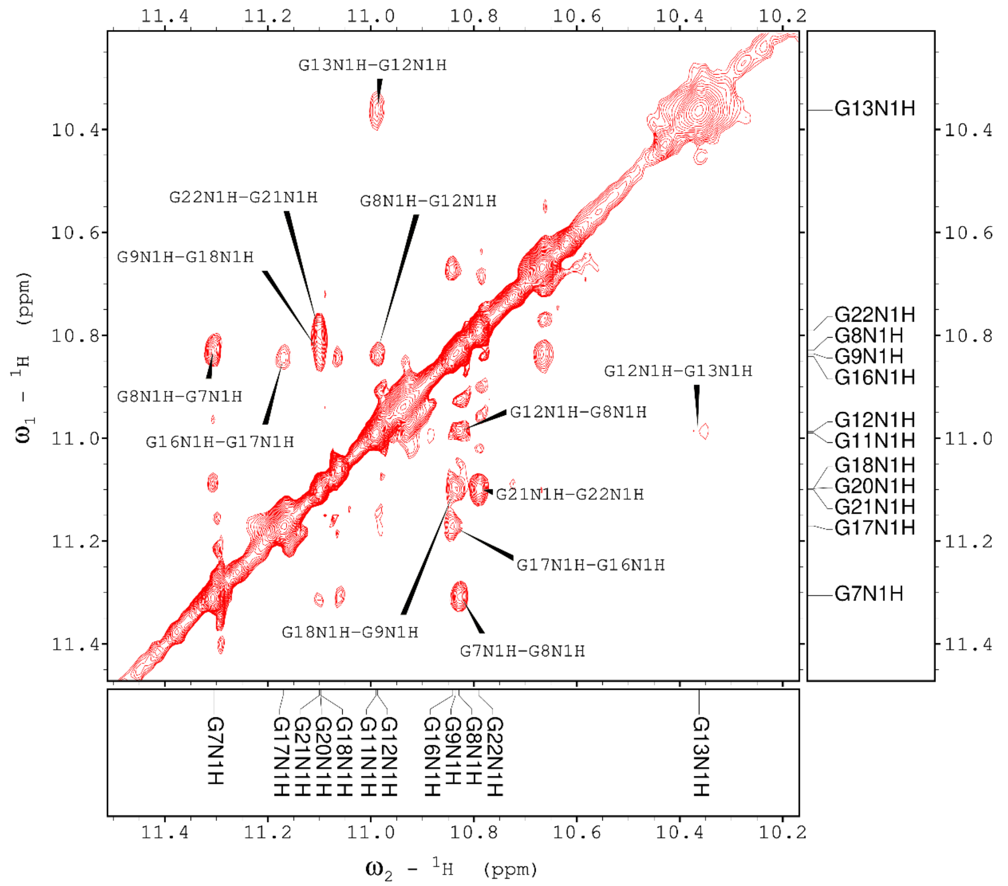

Figure S5. The expanded imine proton region of the 2D-NOESY spectrum of the Pu22:C-1311 1:2 mol/mol complex. Axes are presented in [ppm].

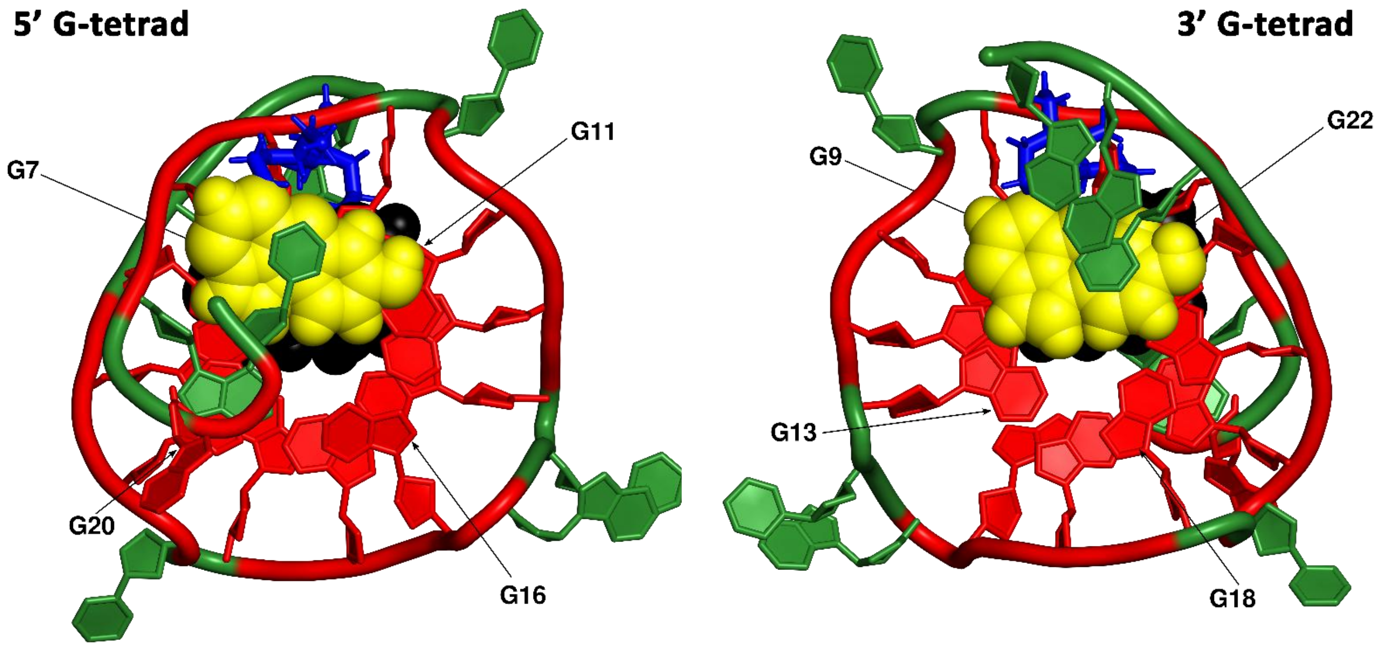

Figure S6. Axial depictions of the Pu22:C-1311 1:2 mol/mol non-covalent complex. Imidazoacridinone ring systems are depicted in yellow, ligand sidechains are depicted in blue, G-tetrad planes are depicted in red, non-G nucleotides are depicted in green.

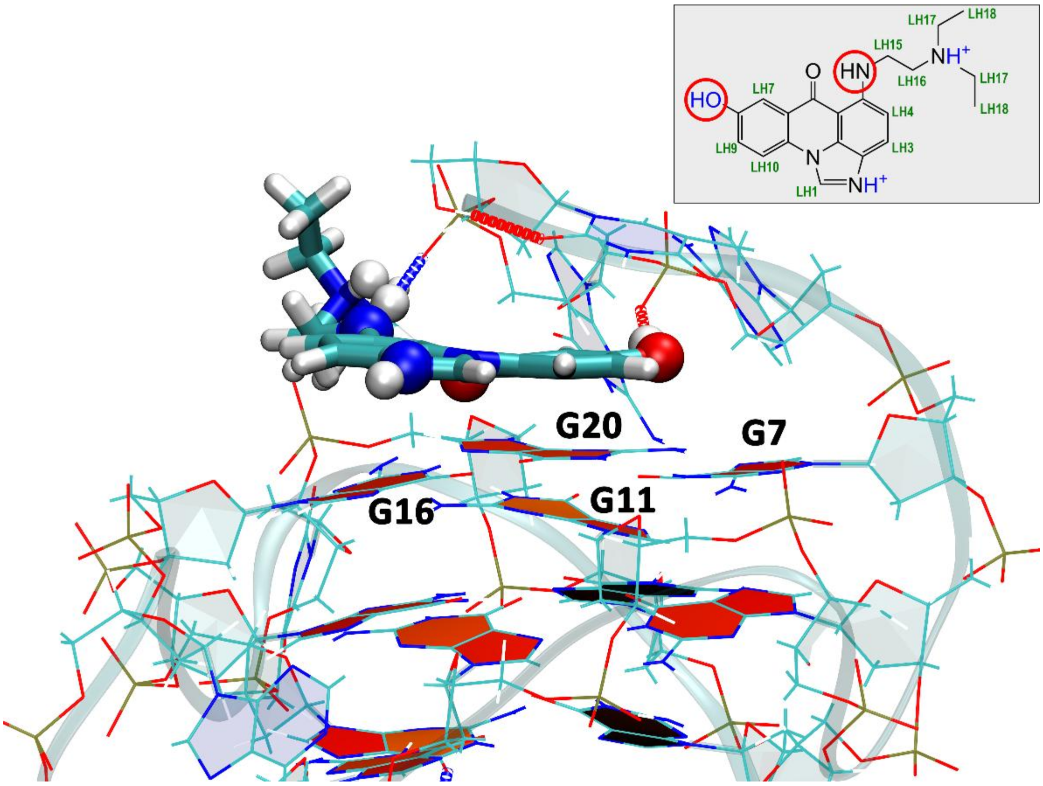

Figure S7. Exemplary hydrogen bonds between C-1311 and Pu22, observed during the restrained MD simulation of the Pu22 1:2 mol/mol non-covalent adduct. The ligand groups participating in the formation of the depicted hydrogen bonds are marked with red circles.

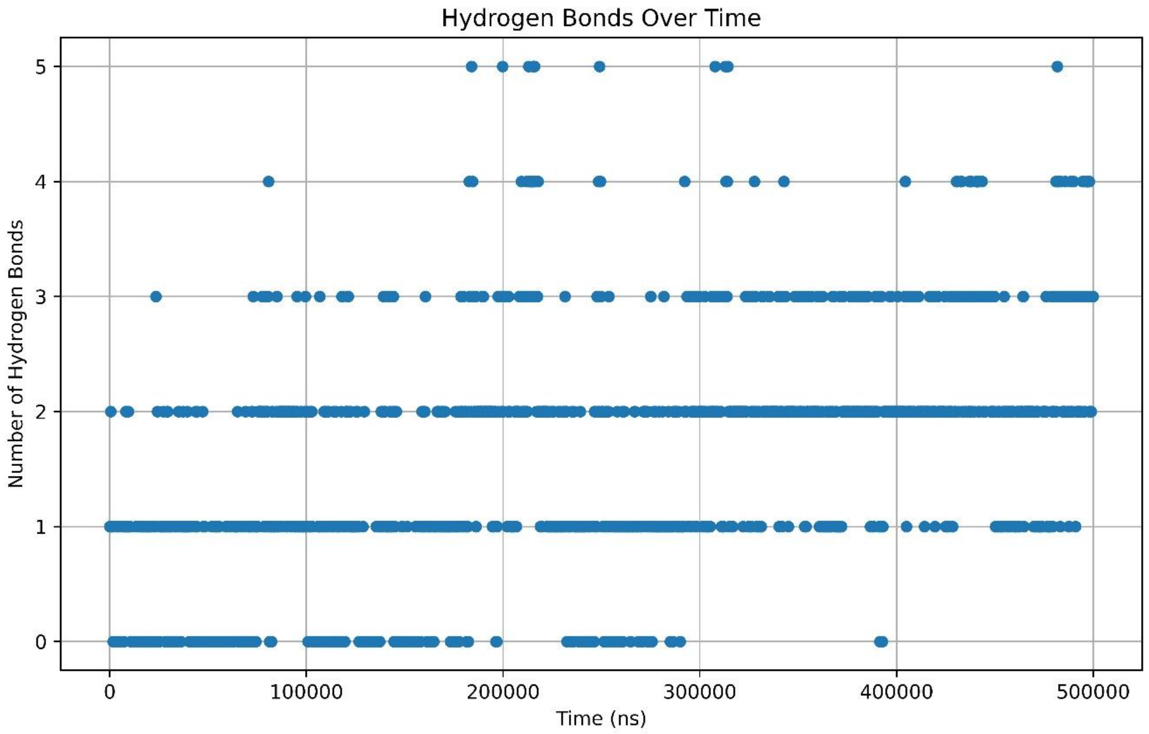

Figure S8. The number of hydrogen bonds formed between C-1311 molecules and the Pu22 host G-quadruplex over the course of the restrained MD simulation.

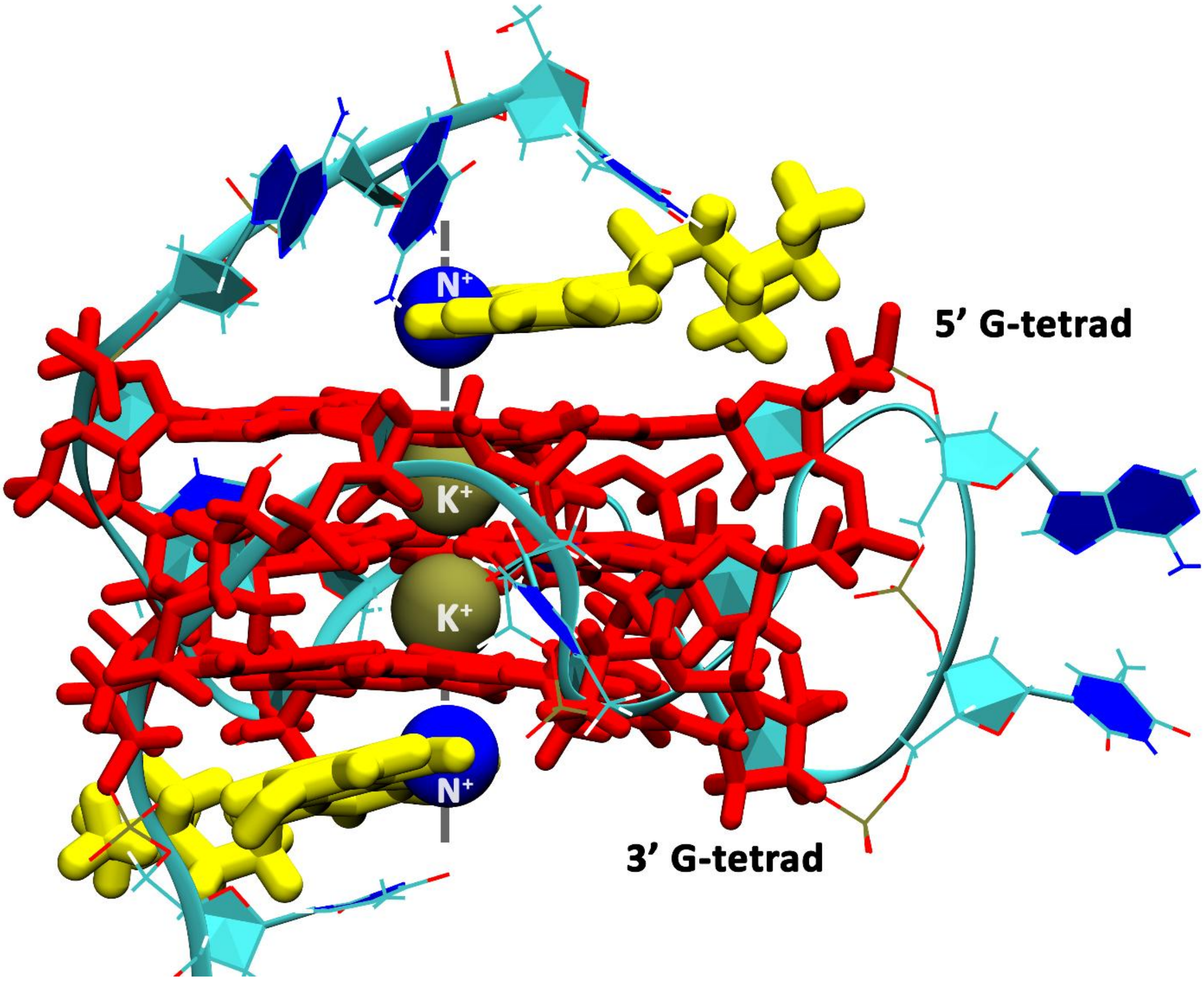

Figure S9. The alignment of two potassium cations (K^+, golden spheres) trapped between the G4-planes and the positively charged nitrogen atoms of the two C-1311 imidazoacridinone ring systems (N^+, blue spheres) along the G-quadruplex axis (dashed line). G-tetrads are depicted in red, ligand molecules are depicted in yellow.

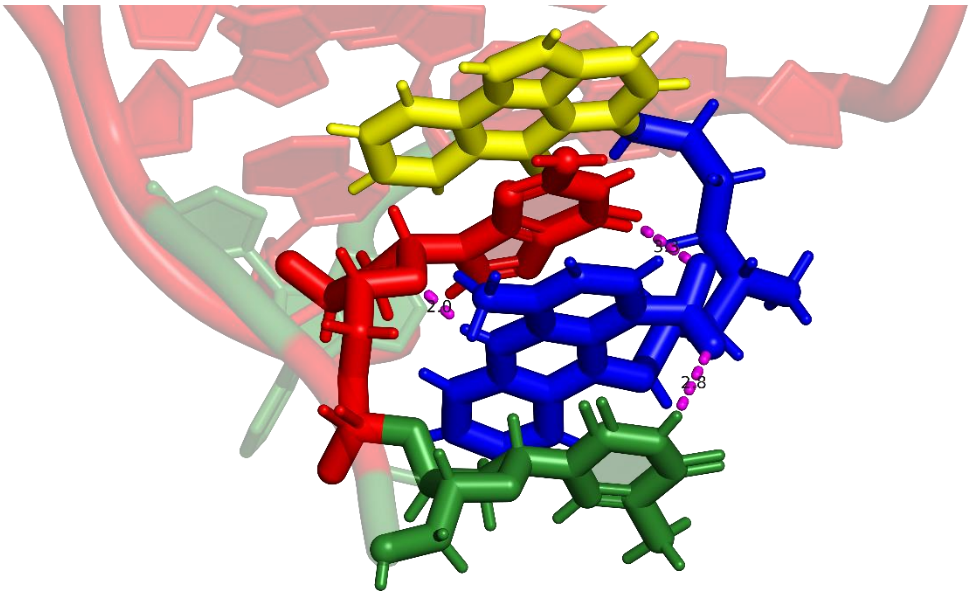

Figure S10. An example of contemporary hydrogen bonds, formed by C-2053 entangled between 5’ G-tetrad od Pu22 and 5’-TGA-3’ flanking residues.

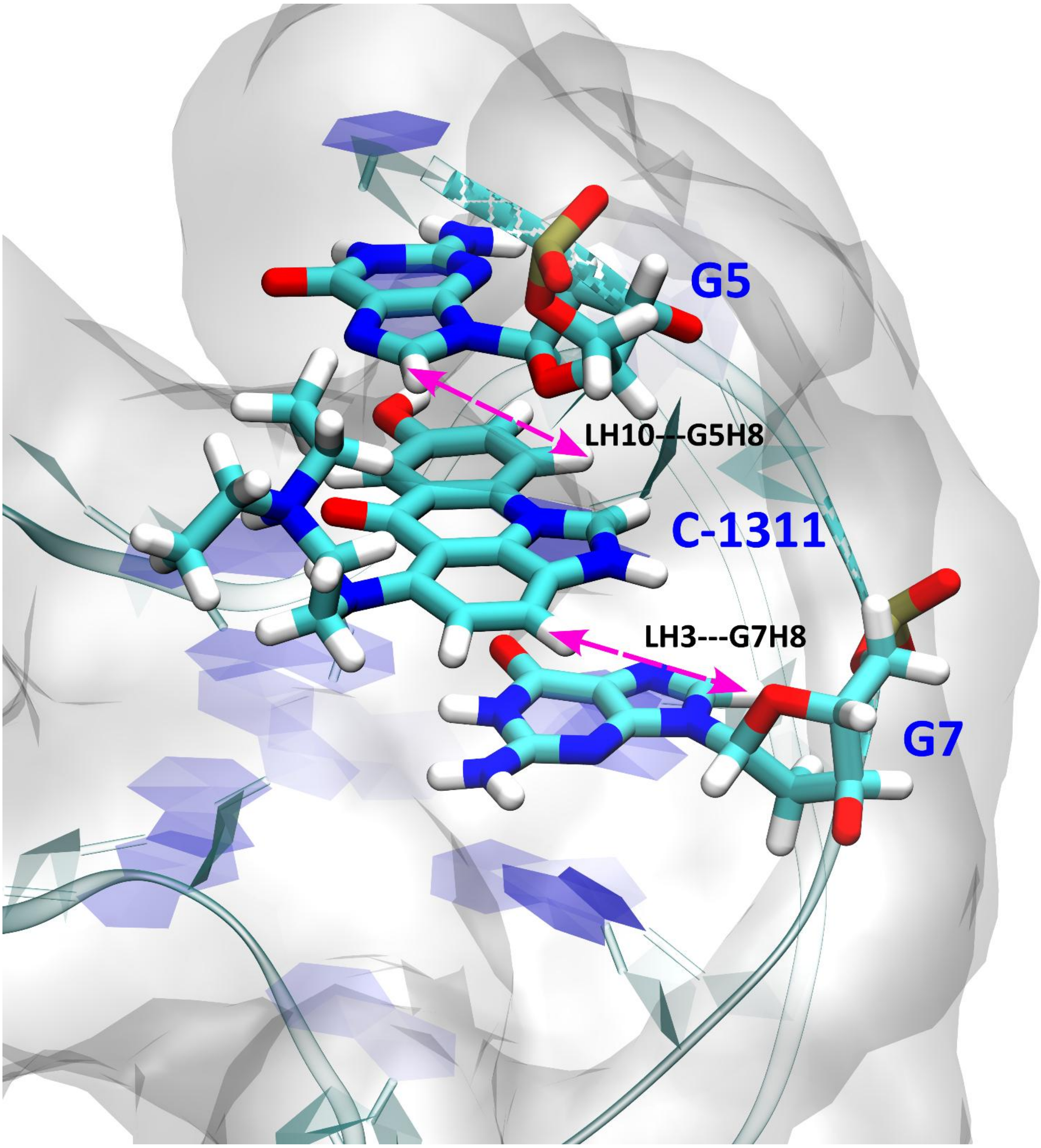

Figure S11. Exemplary Pu22/C-1311 dipolar couplings, observed in 2D NOESY spectrum of Pu22:C-1311 1:2 mol/mol non-covalent adduct.

Table S1. ^{1}H NMR chemical shifts (δ [ppm], referenced to TSP residual signal set to 0.000 ppm) of the protons of the free (uncomplexed) Pu22 G-quadruplex, recorded at 50 °C, H_2O/D_2O 9:1 v/v, 10 mM potassium cacodylate buffer, pH = 5.0, 10 mM KCl. (Continued on the next page.)

Nucleotide	Proton	δ
T4	H1'	6.091
T4	H2'	2.107
T4	H2''	2.078
T4	H3'	4.588
T4	H4'	3.755
T4	H5'	4.147
T4	H5''	3.671
T4	H6	7.511
T4	H7	1.147
G5	H1'	5.542
G5	H2'	2.252
G5	H2''	2.093
G5	H3'	4.590
G5	H4'	3.954
G5	H5'	4.359
G5	H5''	4.302
G5	H8	7.510
A6	H1'	5.760
A6	H2'	2.464
A6	H2''	2.488
A6	H3'	4.737
A6	H4'	3.954
A6	H5'	3.778
A6	H5''	3.610
A6	H8	7.859
G7	H1'	5.923
G7	H2'	2.860
G7	H2''	2.883
G7	H3'	4.867
G7	H4'	4.316
G7	H5'	3.997
G7	H5''	3.949
G7	H8	7.855
G7	N1H	11.691
G8	H1'	6.010
G8	H2'	2.793
G8	H2''	2.771
G8	H3'	4.889
G8	H4'	4.153
G8	H5'	4.400
G8	H5''	4.320
G8	H8	7.552
G8	N1H	11.207
G9	H1'	6.273
G9	H2'	2.459
G9	H2''	2.601
G9	H3'	4.995
G9	H4'	4.161
G9	H5'	4.468
G9	H5''	4.221

Nucleotide	Proton	δ
G9	H8	7.595
G9	N1H	10.662
T10	H1'	6.379
T10	H2'	2.530
T10	H2''	2.542
T10	H3'	4.978
T10	H4'	4.159
T10	H5'	4.466
T10	H5''	4.213
T10	H6	7.733
T10	H7	1.851
G11	H1'	6.000
G11	H2'	2.366
G11	H2''	2.349
G11	H3'	4.966
G11	H4'	4.316
G11	H5'	4.192
G11	H5''	4.118
G11	H8	7.848
G11	N1H	11.663
G12	H1'	6.007
G12	H2'	2.721
G12	H2''	2.700
G12	H3'	4.961
G12	H4'	4.320
G12	H5'	4.131
G12	H5''	4.074
G12	H8	7.759
G12	N1H	11.474
G13	H1'	6.286
G13	H2'	2.479
G13	H2''	2.614
G13	H3'	4.915
G13	H4'	4.149
G13	H5'	4.358
G13	H5''	4.156
G13	H8	7.715
G13	N1H	11.033
T14	H1'	5.680
T14	H2'	1.918
T14	H2''	1.949
T14	H3'	4.293
T14	H4'	3.688
T14	H5'	3.346
T14	H5''	3.334
T14	H6	7.094
T14	H7	1.535
A15	H1'	6.545
A15	H2'	2.824
A15	H2''	2.949

Nucleotide	Proton	δ
A15	H3'	5.048
A15	H4'	3.611
A15	H5'	4.153
A15	H5"	4.071
A15	H8	8.394
G16	H1'	6.031
G16	H2'	2.819
G16	H2"	2.841
G16	H3'	4.894
G16	H4'	4.020
G16	H5'	4.319
G16	H5"	4.122
G16	H8	7.954
G16	N1H	11.868
G17	H1'	6.067
G17	H2'	2.823
G17	H2"	2.848
G17	H3'	4.894
G17	H4'	4.409
G17	H5'	4.245
G17	H5"	4.174
G17	H8	7.656
G17	N1H	11.216
G18	H1'	6.309
G18	H2'	2.467
G18	H2"	2.485
G18	H3'	5.002
G18	H4'	3.962
G18	H5'	4.119
G18	H5"	4.078
G18	H8	7.656
G18	N1H	10.973
T19	H1'	6.087
T19	H2'	2.338
T19	H2"	2.318
T19	H3'	4.591
T19	H4'	3.755
T19	H5'	4.291
T19	H5"	4.135
T19	H6	7.507
T19	H7	1.805
G20	H1'	5.866
G20	H2'	2.647
G20	H2"	2.674
G20	H3'	4.938
G20	H4'	4.051
G20	H5'	4.247

Nucleotide	Proton	δ
G20	H5"	4.175
G20	H8	7.660
G20	N1H	11.248
G21	H1'	5.904
G21	H2'	2.539
G21	H2"	2.591
G21	H3'	4.938
G21	H4'	4.100
G21	H5'	4.122
G21	H5"	4.042
G21	H8	7.753
G21	N1H	11.333
G22	H1'	6.050
G22	H2'	2.639
G22	H2"	2.660
G22	H3'	4.854
G22	H4'	4.377
G22	H5'	4.170
G22	H5"	4.095
G22	H8	7.474
G22	N1H	11.035
T23	H1'	5.783
T23	H2'	2.204
T23	H2"	2.228
T23	H3'	4.620
T23	H4'	4.052
T23	H5'	4.021
T23	H5"	3.942
T23	H6	7.042
T23	H7	1.420
A24	H1'	5.662
A24	H2'	2.258
A24	H2"	2.283
A24	H3'	4.465
A24	H4'	4.056
A24	H5'	3.846
A24	H5"	3.630
A24	H8	7.683
A25	H1'	5.553
A25	H2'	2.195
A25	H2"	2.098
A25	H3'	4.270
A25	H4'	3.756
A25	H5'	3.617
A25	H5"	3.382
A25	H8	7.446

Table S2. ^{1}H NMR chemical shifts (δ [ppm], referenced to TSP residual signal set to 0.000 ppm) of the protons of the Pu22:C-1311 1:2 mol/mol complex, recorded at 50 °C, H_2O/D_2O 9:1 v/v, 10 mM potassium cacodylate buffer, pH = 5.0, 10 mM KCl. (Continued on the next page.)

Nucleotide	Proton	δ
-	LH1	8.082
-	LH3	7.112
-	LH4	5.978
-	LH7	6.696
-	LH9	6.486
-	LH10	7.079
	LH15	3.112
-	LH16	3.007
-	LH17	2.937
-	LH18	1.055
T4	H1'	6.103
T4	H2'	2.135
T4	H2''	2.350
T4	H3'	4.567
T4	H6	7.518
G5	H1’	5.884
G5	H8	8.020
A6	H1'	6.087
A6	H2'	2.521
A6	H2''	2.639
A6	H3'	4.778
A6	H8	8.151
G7	H1'	5.888
G7	H2'	2.608
G7	H2''	2.844
G7	H3'	4.869
G7	H8	7.893
G7	N1H	11.306
G8	H1'	5.973
G8	H2'	2.478
G8	H2''	2.758
G8	H3'	4.881
G8	H8	7.526
G8	N1H	10.829
G9	H1'	6.200
G9	H2'	2.453
G9	H2''	2.536
G9	H3'	5.008
G9	H8	7.549
G9	N1H	10.836
T10	H1'	6.371
T10	H2’	2.335
T10	H2”	2.537
T10	H3'	4.970
T10	H6	7.729
G11	H1'	5.952
G11	H2'	2.249
G11	H2''	2.705
G11	H3'	4.928
G11	H8	7.751
G11	N1H	10.990

Nucleotide	Proton	δ
G12	H1'	5.872
G12	H2'	2.433
G12	H2''	2.625
G12	H3'	4.907
G12	H8	7.587
G12	N1H	10.987
G13	H1'	6.252
G13	H2'	2.585
G13	H2''	2.659
G13	H3'	4.929
G13	H8	7.568
G13	N1H	10.362
T14	H1'	5.526
T14	H2'	1.252
T14	H2''	1.729
T14	H3'	4.460
T14	H6	6.934
A15	H1'	6.578
A15	H2'	2.831
A15	H2''	2.950
A15	H3'	5.056
A15	H8	8.471
G16	H1'	5.961
G16	H2'	2.398
G16	H2''	2.750
G16	H3'	4.831
G16	H8	7.825
G16	N1H	10.841
G17	H1'	5.971
G17	H2'	2.477
G17	H2''	2.752
G17	H3'	4.857
G17	H8	7.511
G17	N1H	11.171
G18	H1'	6.284
G18	H2'	2.497
G18	H2''	2.621
G18	H3'	5.019
G18	H8	7.592
G18	N1H	11.098
T19	H1'	6.386
T19	H2’	2.355
T19	H2”	2.537
T19	H3'	4.968
T19	H6	7.728
G20	H1'	5.880
G20	H2'	2.217
G20	H2''	2.663
G20	H3'	4.942
G20	H8	7.722
G20	N1H	11.098

Nucleotide	Proton	δ
G21	H1'	5.895
G21	H2'	2.540
G21	H2''	2.664
G21	H3'	4.943
G21	H8	7.698
G21	N1H	11.100
G22	H1'	6.083
G22	H2'	2.429
G22	H2''	2.667
G22	H3'	4.851
G22	H8	7.451
G22	N1H	10.791
T23	H1'	5.805
T23	H2'	1.889
T23	H2''	2.125
T23	H3'	4.656
T23	H6	7.110

Nucleotide	Proton	δ
A24	H1'	5.792
A24	H2'	2.199
A24	H2''	2.244
A24	H3'	4.557
A24	H8	7.905
A25	H1'	5.599
A25	H2'	2.151
A25	H2''	2.032
A25	H3'	4.587
A25	H8	7.627

Table S3. The definition of distance restraints used in molecular dynamics simulations. When the distance between two protons fits between ‘down’ and ‘up1’ values: no potential is being used to bring the respective protons closer; when the distance fits between ‘up1’ and ‘up2’ or its value is lower than ‘down’: a quadratic potential is being added; when the distance exceeds ‘up2’ limit: the potential becomes linear.

Distance restraint definition:	down [nm]	up1 [nm]	up2 [nm]
**medium**	0.2000	0.4000	0.5000
**weak**	0.3000	0.5000	0.5500
**very weak**	0.3000	0.6000	0.6500
